# Supplementary material for: Effectiveness of Multimodal imaging for the Evaluation of Retinal oedema And new vesseLs in Diabetic retinopathy (EMERALD)
Source: BMJ Open. 2019 Jun 28;9(6):e027795. doi: 10.1136/bmjopen-2018-027795 (PMC6609061; doi:10.1136/bmjopen-2018-027795)
Supplement: Author's manuscript [file bmjopen-2018-027795.draft_revisions.pdf]

BMJ Open is committed to open peer review. As part of this commitment we make the peer review history of every article we publish publicly available.

When an article is published we post the peer reviewers' comments and the authors' responses online. We also post the versions of the paper that were used during peer review. These are the versions that the peer review comments apply to.

The versions of the paper that follow are the versions that were submitted during the peer review process. They are not the versions of record or the final published versions. They should not be cited or distributed as the published version of this manuscript.

BMJ Open is an open access journal and the full, final, typeset and author-corrected version of record of the manuscript is available on our site with no access controls, subscription charges or pay-per-view fees (http://bmjopen.bmj.com).

If you have any questions on BMJ Open's open peer review process please email info.bmjopen@bmj.com

BMJ Open

Effectiveness of Multimodal imaging for the Evaluation of Retinal oedema And new vesseLs in Diabetic retinopathy (EMERALD): Protocol for a Diagnostic Study

Journal:	*BMJ Open*
Manuscript ID	bmjopen-2018-027795
Article Type:	Protocol
Date Submitted by the Author:	19-Nov-2018
Complete List of Authors:	Lois, Noemi; Wellcome-Wolfson Institute for Experimental Medicine, Ophthalmology<br>Cook, Jonathan; University of Oxford, NDORMS<br>Aldington, Stephen; Gloucester Hospitals NHS Foundation Trust<br>Waugh, Norman; University of Warwick, Warwick Evidence<br>Mistry, Hema; University of Warwick, Warwick Evidence<br>Sones, William; University of Oxford, Centre for Statistic in Medine, Oxford, UK OX37LD 01865223450<br>McAuley, Danny; Queen's University Belfast, Centre for Infection and Immunity<br>Aslam, Tariq; University of Manchester,<br>Bailey, Claire; University of Bristol<br>Chong, Victor; Royal Free Hospital NHS Foundation Trust, Ophthalmology<br>Ghanchi, Faruque; Bradford Royal Infirmary, Ophthalmology<br>Scanlon, Peter; Gloucestershire Hospitals NHS Foundation Trust<br>Sivaprasad, Sobha; Consultant Ophthalmologist, Laser and Retinal Research Unit<br>Steel, David; Sunderland Eye Infirmary; Institute of Genetic Medicine, Newcastle University<br>Styles, Caroline; Queen Margaret Hospital, Ophthalmology<br>McNally, Christine; Northern Ireland Clinical Trials Unit<br>Rice, Rachael ; Northern Ireland Clinical Trials Unit<br>Prior, Lindsay; Queens University Belfast<br>Azuara-Blanco, Augusto ; Queens University Belfast , Wellcome-Wolfson Institute for Experimental Medicine
Keywords:	Diabetic retinopathy < DIABETES & ENDOCRINOLOGY, HEALTH ECONOMICS, Medical retina < OPHTHALMOLOGY, PUBLIC HEALTH, QUALITATIVE RESEARCH

SCHOLARONE™
Manuscripts

Effectiveness of **M**ultimodal imaging for the **E**valuation of **R**etinal oedema **A**nd new vesse**L**s in **D**iabetic retinopathy (EMERALD): Protocol for a Diagnostic Study

Noemi Lois, MD[1], PhD, FRCS(Ed), FRCOphthal
Jonathan Cook, PhD, BSc Hons[2]
Stephen Aldington, PGDip, HND[3]
Norman Waugh, MRCP(UK), MPH[4]
Hema Mistry PhD[4]
William Sones[2]
Danny McAuley MD, FRCP[1, 5]
Tariq M Aslam, DM (Oxon), FRCSEd, PhD[6]
Claire Bailey, MD, BM BCh, MA[7]
Victor Chong, MD, MPHIL, MB ChB[8]
Faruque DaudBhai Ghanchi, MBBS[9]
Peter H Scanlon, MD, FRCP, FRCOphth[3]
Sobha Sivaprasad, DM, FRCS, FRCOphth[10]
David H Steel, MBBS, MD, FRCOphth[11]
Caroline Styles, MB ChB[12]
Christine McNally[13]
Rachael Rice[13]
Lindsay Prior, PhD[14]
Augusto Azuara-Blanco, MD, PhD, FRCS(Ed), FRCOphthal[14]
On behalf of the EMERALD Study Group

From The Wellcome-Wolfson Institute for Experimental Medicine,[1] Queen's University, Belfast; Centre for Statistics in Medicine, University of Oxford;[2] Gloucestershire Hospitals NHS Foundation Trust; [3] The Division of Health Sciences, University of Warwick;[4] The Regional Intensive Care Unit, Royal Victoria Hospital, Belfast;[5] The Manchester Academic Health Science Centre, Manchester Royal Eye Hospital and Division of Pharmacy and Optometry, School of Health Sciences, Faculty of Biology, Medicine and Health, University of Manchester, Manchester;[6] Bristol Eye Hospital, Bristol;[7] Royal Free Hospital NHS Foundation Trust, London;[8] Bradford Teaching Hospitals, Bradford Royal Infirmary, Bradford;[9] NIHR Moorfields Biomedical Research Centre, London;[10] Sunderland Eye Infirmary, Sunderland and Institute of Genetic Medicine, Newcastle University, Newcastle Upon Tyne;[11] Queen Margaret Hospital, Fife, Scotland;[12] The Northern Ireland Clinical Trials Unit (NICTU), Belfast;[13] and the Centre for Public Health, Queens University, Belfast.[14]

Correspondence: Prof. Noemi Lois, Wellcome-Wolfson Institute for Experimental Medicine, Queen's University Belfast, 97 Lisburn Road, BT9 7BL Belfast, UK, n.lois@qub.ac.uk, Telephone: 00 44 28 9097 6462

Trial Registration: ISRCTN Number: 10856638 (registered on 8/03/2017)

Protocol Version: 3 (March 20, 2018). The full protocol, study guidelines and patient related materials can be accessed at the EMERALD website (http://www.nictu.hscni.net/emerald-trial-/)

Key words: Diabetes, Diabetic Macular Oedema, Diabetic Macular Edema, DMO, PDR, Pathway, laser, anti-VEGFs, cost-effectiveness.

This protocol was prepared following SPIRIT and STARD guidelines; all items listed in the World Health Organisation Trial Registration Data Set have been specified throughout this protocol.

For peer review only

ABSTRACT (250 words)

Introduction
Diabetic macular oedema (DMO) and proliferative diabetic retinopathy (PDR) are the major causes of sight loss in people with diabetes. Due to the increased prevalence of diabetes, the workload related to these complications is increasing making it difficult for Hospital Eye Services (HSE) to meet demands.

Methods and analysis
EMERALD is a prospective, case-referent, cross-sectional diagnostic study which aims at determining the diagnostic performance, cost-effectiveness and acceptability of a new form of surveillance for people with stable DMO and/or PDR which entails multimodal imaging and image review by an ophthalmic grader, using the current standard of care (evaluation of patients in clinic by an ophthalmologist) as the reference standard. If safe, cost-effective and acceptable, this pathway could help HES by freeing ophthalmologist time. The primary outcome of EMERALD is sensitivity of the new surveillance pathway in detecting active DMO/PDR. Secondary outcomes include specificity, concordance between new and the standard care pathway, positive and negative likelihood ratios, cost-effectiveness, acceptability, proportion of patients requiring subsequent full clinical assessment, unable to undergo imaging, with inadequate quality images or indeterminate findings.

Ethics and dissemination
Ethical approval was obtained for this study from the Office for Research Ethics Committees Northern Ireland (reference 17/NI/0124). Study results will be published as a Health Technology Assessment monograph, in peer-reviewed national and international journals and presented at national/international conferences and to patient groups.

Registration details
ISRCTN: 10856638 (date of registration 08/03/2017)

Strengths and limitations of study (80 words)

Strengths:
- Prospective, multicenter study
- Masking of graders to results of the reference standard and masking of ophthalmologists to results of the grader's pathway.
- Evaluation of the acceptability of the new pathway to patients and health professionals
- State of the art diagnostic imaging technologies used
- Early involvement in all aspects of the study of patients and public
- Outcome measures of importance to patients

Limitations:
- Lack of fluorescein angiography to confirm active proliferative diabetic retinopathy.

Introduction (1000 words)

For peer review only

Diabetic Retinopathy (DR) is the most common microvascular complication of diabetes and a leading cause of visual loss among individuals of working age (1). Patients with DR may lose sight as a result of the development of diabetic macular oedema (DMO) and/or proliferative diabetic retinopathy (PDR), the major complications of DR. In the former, fluid accumulates in the central part of the retina, the macula, which is responsible for detailed central vision. In the latter, abnormal new blood vessels ("new vessels") grow on the optic nerve head or on the surface of the retina and towards the inside of the eye (the vitreous cavity) leading to sight loss from haemorrhaging or/and traction on the retina with subsequent retinal detachment. Due to the increasing numbers of people with diabetes, it is expected that the burden of DR will continue to rise. Indeed, it has been estimated that the worldwide prevalence of DR will increase from 126.6 million in 2010 to 191 million by 2030 (2).

The prevalence of DMO in England was estimated to be 7% of the total diabetic population in 2010 (3). A very similar estimate of the prevalence was found in an individual participant data meta-analysis, which included 22,896 individuals from 35 studies, conducted in Asia, Australia, Europe, and US, which provided an overall age-standardised prevalence of DMO of 6.8% (4). Based on this published prevalence of DMO and considering the prevalence of diabetes in the UK (https://www.diabetes.org.uk) it can be estimated that there are 280,000 people in the UK affected by DMO. In the UK, patients with DMO are treated with focal or grid macular laser [when the central retinal thickness (CRT), measured by means of spectral domain optical coherence tomography (SD-OCT), is <400 microns], which is delivered in a single session, or with injections into the eye of anti-vascular endothelial growth factor (anti-VEGF) therapies, currently in the National Health Service (NHS) ranibizumab (Lucentis) and aflibercept (Eylea) [when the central retinal thickness measured by SD-OCT is ≥400 microns] (www.nice.org.uk/guidance/ta274; www.nice.org.uk/guidance/ta346). Intraocular steroids are also available for patients that do not respond to the above therapies and are pseudophakic (i.e. have had their cataracts removed) (https://www.nice.org.uk/guidance/ta301). Once treated, long-term follow-up is required, to determine whether DMO recurs. Typically, patients are followed every three to four months following laser treatment for DMO or monthly initially and then every 1-3 months thereafter following treatment with anti-VEGFs (https://www.rcophth.ac.uk/wp-content/uploads/2014/12/2013-SCI-301-FINAL-DR-GUIDELINES-DEC-2012-updated-July-2013.pdf). Follow-up continues for the rest of the patient's life.

The estimated prevalence of PDR in the individual participant data meta-analysis referred to above (4) was 6.96%; based on the prevalence of diabetes in UK (https://www.diabetes.org.uk) there are ~278,400 people in the UK affected by PDR. At present in the UK, patients with PDR are treated with panretinal laser photocoagulation (PRP), which is delivered most often in two to three sessions. Once treatment is completed, patients are followed at 4-6 month intervals for their lifetime to determine whether reactivation occurs, as new vessels in PDR could come back and tractional retinal detachment can still ensue (https://www.rcophth.ac.uk/wp-content/uploads/2014/12/2013-SCI-301-FINAL-DR-GUIDELINES-DEC-2012-updated-July-2013.pdf). A high proportion of patients with DR followed in Hospital Eye Services (HES) have treated and inactive PDR (5).

For peer review only - http://bmjopen.bmj.com/site/about/guidelines.xhtml

Currently in the NHS ophthalmologists with expertise in retinal diseases assess patients during follow up visits. At each visit, patients with DMO are evaluated with a visual acuity test, most often undertaken by a nurse; SD-OCT, obtained by a photographer and interpreted by the ophthalmologist, and slit-lamp biomicroscopy, undertaken by an ophthalmologist, who then determines whether DMO is present or absent. SD-OCT is a non-invasive, user-friendly and safe imaging technique that obtains scans of the back of the eye. SD-OCT allows measurement of the CRT (which is often increased when DMO is present) and visualising fluid in the retina which is the hallmark of DMO. SD-OCT has been extensively used in clinical trials and clinical practice to determine the presence of DMO, select treatment, and monitor the response to treatment (6-12). In the follow-up of patients with PDR ophthalmologists typically examine the patient by slit-lamp biomicroscopy. Photographs are not routinely obtained in clinic to determine whether or not active PDR is present. Standard cameras are not able to image the whole retina. It is possible, however, to obtain several images to capture the appearance of the centre, superior and inferior parts of the retina; the retinal periphery cannot be imaged with standard cameras. In recent years new ultra-wide field imaging has become available, allowing imaging of the entire retina. This technology may be, thus, preferable, and it may be easier for patients and reduce the time required to obtain images.

Given the high number of people with DMO and PDR, the need for patients to be seen at short follow-up intervals, the need for frequent treatments and the requirement for long-term follow-up, there is a very large workload in HES related to DMO/PDR. This is making it difficult for the NHS to cope with the demand, in particular, due to shortage of ophthalmologists. This is only expected to get worse given the increasing prevalence of diabetes. Identifying new ways of increasing the NHS capacity and efficiency without compromising quality of care would greatly benefit the NHS.

The purpose of EMERALD is to determine whether successfully treated patients with DMO/PDR could be followed without a face-to-face examination by an ophthalmologist. EMERALD will evaluate a new care pathway which will include multimodal retinal imaging and separate image assessment by trained ophthalmic graders. This new pathway will be compared to the current standard care pathway which is, for DMO, having an ophthalmologist evaluating patients in clinic by slit-lamp biomicroscopy and with access to SD-OCT images and for PDR evaluating patients in clinic by slit-lamp biomicroscopy. EMERALD will determine how accurate the new pathway is at determining which patients have active or inactive disease when compared with the standard of care and its costs and acceptability by patients and health professionals.

<u>Methods and analysis (3500 words)</u>

1. <u>Study design and setting</u>

Prospective, diagnostic study of patients with DMO, PDR, or both, who had been previously successfully treated and who, at the time of enrolment in the study, may have active or inactive disease (both are required to evaluate the diagnostic performance of the new pathway).

For peer review only - http://bmjopen.bmj.com/site/about/guidelines.xhtml

EMERALD has a case-referent cross-sectional diagnostic study design with both sampling (selection) of patients and data collection carried out prospectively (13). This approach provides both a cost-efficient study design and low risk of bias in terms of diagnostic accuracy (14).

EMERALD is set within specialised HES in the UK. All participating centres have extensive experience with the management of DMO and PDR.

A Patient and Public Involvement (PPI) group was established early on in the study, at the stage of trial conception. The PPI group provided input on study design, outcome measures, and helped with patient-related materials.

2. Participants: Eligibility criteria

Inclusion Criteria:

Adults (18 years of age or older) with type 1 or 2 diabetes with previously successfully treated DMO and/or PDR in one or both eyes and in whom, at the time of enrolment in the study, DMO and/or PDR may be active or inactive. Patients can be recruited only once.

Active DMO is defined as CRT of > 300 microns and/or presence of intraretinal/subretinal fluid on SD-OCT due to DMO.

Inactive DMO is defined as no intraretinal/subretinal fluid.

Active PDR is defined by the presence of sub-hyaloid/vitreous haemorrhage and/or active new vessels (new vessels with lack of fibrosis on them)

Inactive PDR is defined by the lack of preretinal/vitreous haemorrhage and lack of active new vessels.

Exclusion Criteria:

1) Unable to provide informed consent
2) Patients who do not read, speak or understand English.

3. Outcome measures

Primary outcome:

• Sensitivity of the new pathway (ophthalmic grader pathway) in detecting active DMO/PDR, using the standard care pathway as the reference standard.

Secondary Outcomes:

• Specificity, concordance (agreement) between new pathway (ophthalmic grader pathway) and the standard care pathway, positive and negative likelihood ratios

For peer review only - http://bmjopen.bmj.com/site/about/guidelines.xhtml

For peer review only

• Cost-effectiveness
• Acceptability
• Proportion of patients requiring subsequent full clinical assessment
• Proportions of patients unable to undergo imaging, with inadequate quality images or indeterminate findings.

4. Study Procedures and Schedule of Assessments

Ophthalmic Grader Pathway and Standard Care Pathway

In EMERALD a new clinical care pathway (Figure 1), the ophthalmic grader pathway, will be evaluated and compared with the current standard of care.

If this new pathway were to be used in clinical practice, patients would undergo multimodal retinal imaging with subsequent review by trained ophthalmic graders. If active disease were to be detected or if the ophthalmic grader were to be uncertain as to whether or not there was active DMO/PDR present, the patient would be referred to an ophthalmologist for a full assessment. If the patient were to be stable (i.e. inactive DMO/PDR) the patient would remain under this model of surveillance with a pre-determined interval.

For the purpose of EMERALD, all patients will go through the standard care pathway which is: 1) for DMO: ophthalmologist evaluating patients in clinic by slit-lamp biomicroscopy and with access to SD-OCT images, which are routinely obtained for the evaluation of DMO in clinic; 2) for PDR: ophthalmologists evaluating patients in clinic by slit-lamp biomicroscopy.

Selection of Ophthalmic Graders and Training

Currently, ophthalmic photographers/imaging technicians obtain images and interpret them routinely, but make no decisions on the care of patients. In ophthalmic services, there are also ophthalmic graders that have been trained to interpret findings on fundus images for the purpose of undertaking DR screening.

For EMERALD the ophthalmic graders at each participating site will be selected as follows. First, local principal investigators (PIs) would provide names of individuals they believe have experience obtaining and/or grading images of patients with DMO and PDR; these individuals would be also confirming their interest and willingness to participate in EMERALD. It is possible that some ophthalmic graders selected for EMERALD will be already involved in the grading of images for DR screening.

Graders identified by the PIs will be asked to fill out questionnaires detailing their experience imaging/grading DMO/PDR, recognising features of DMO/PDR, and whether they feel confident identifying DMO on SD-OCT and new vessels on fundus images. Graders stating they do not have experience imaging/grading DMO/PDR and/or those stating they could not recognise features of DMO/PDR will not be invited to take part in EMERALD as graders.

For peer review only - http://bmjopen.bmj.com/site/about/guidelines.xhtml

Formal training, during a training meeting(s) in which features of active/inactive DMO/PDR will be reviewed and discussed and where extensive clinical examples will be presented, will be provided to all EMERALD ophthalmic graders prior to the initiation of the study, as described in the EMERALD Study Manual.

To ensure graders selected will be in a position to undertake the task of grading images, all potential graders will be required to take a test involving the reading of OCTs and fundus images. Only those graders who reach a minimum of 80% of correct answers to detect presence of DMO or active PDR, when present, will be invited to act as graders. Graders will be allowed to undergo further training and take the test a second time but if the minimum of correct answers is not reached they will not be able to act as graders.

A web-based teaching module with examples of DMO/PDR will be prepared so that graders can access it to consolidate their knowledge. Clear guidelines on when patients would need to be referred for an assessment by an ophthalmologist will be also given.

Schedule of Assessments for EMERALD

Patient flow and procedures undertaken in EMERALD have been summarised in Figure 2.

Written informed consent will be obtained by ophthalmologists/deputes from all participants prior to any study procedures being undertaken.

The following information obtained during the standard care pathway will be recorded in the Case Report Forms (CRFs):

• Medical and ophthalmic history.
• Best-corrected visual acuity.
• Whether there was active or inactive DMO / PDR and whether or not there were features that could make imaging difficult (e.g. small pupil).
• Details on presence/absence and location of active/inactive new vessels in the disc and elsewhere in the retina and/or pre-retinal haemorrhage/vitreous haemorrhage
• Information on the proposed plan for the patient (review/treatment)

Patients will undergo ETDRS 7 field and ultra-wide field imaging, obtained following the guidelines set in the EMERALD Study Manual and will complete EQ-5D 5L, NEI VFQ-25 and VisQoL questionnaires.

Patients willing to participate in the Focus Group Discussions will be consented; a group of them will be contacted at a later date for this purpose (see below).

Fundus photographs and SD-OCTs will be anonymised and transferred to Queens' University Belfast (QUB) reading centre where they will be uploaded in an electronic website developed for the study; these anonymised images will be then made accessible to the ophthalmic graders (and ophthalmologists determining the "enhanced reference standard" for PDR, see below) for the purpose of grading.

For peer review only - http://bmjopen.bmj.com/site/about/guidelines.xhtml

Ophthalmic graders will determine (and record in the appropriate CRF):
• Whether there is active/inactive DMO/PDR (or if unsure)
• Location of new vessels
• Whether patient could continue review in the ophthalmic grader pathway or requires assessment by an ophthalmologist and the reasons why.

Enhanced Reference Standard

The reference standard for PDR (ophthalmologist evaluating patients in clinic by slit-lamp biomicroscopy) will be used for the primary analysis. The reference standard for PDR, however, could potentially be improved. There is a possibility that new vessels (indicative of PDR) may not be seen by the ophthalmologist on slit-lamp biomicroscopy but could be detected in a fundus photograph. In order to determine the impact of this potential event EMERALD will also evaluate an alternative "enhanced" reference standard which will consist of the ophthalmologist assessment supplemented by the evaluation of the fundus images (7 field ETDRS and ultra-wide field fundus images) done by an ophthalmologist. This reading by the ophthalmologist of the fundus images will be done only after the reference standard has been set, to ensure it will not affect or influence the reference standard. If either, slit-lamp biomicroscopy, 7 field ETDRS or ultra-wide angle fundus images detect active PDR, the patient will be considered to have "active" PDR under this "enhanced" reference standard. This information will be recorded in the appropriate CRF. The PDR status based upon the enhanced reference standard will be used in a sensitivity analysis of the new pathway's diagnostic accuracy.

Masking

Ophthalmic graders will be masked to the reference standard; to ensure this, they will not interpret images from patients recruited at their own centre and will not have access to results of the reference standard. They will not read 7 field ETDRS and wide-angle fundus images or SD-OCTs of the same eye either, to ensure their reading of one imaging technology will not influence their reading of the other.

Ophthalmologists doing the standard of care evaluation will also be masked to the findings/decisions made by the ophthalmic graders (who will be reviewing the images at a later date).

Ophthalmologists determining the "enhanced" reference standard will be masked in the same manner as the ophthalmic graders.

Patients will also be masked to findings/decisions made by the ophthalmic graders (these will not be available at the time of the study's clinical visit).

5. <u>Data collection and quality checks</u>

CRFs will be used to collect data. Monitoring during the study will check the accuracy of entries on CRF's against source documents, adherence to protocol, procedures and to the International Conference of Harmonisation Good Clinical Practice (ICH-GCP) guidelines and regulatory requirements. Monitoring visits will

For peer review only - http://bmjopen.bmj.com/site/about/guidelines.xhtml

be undertaken by a monitor from the Northern Ireland Clinical Trials Unit (NICTU). To ensure accurate, complete and reliable data are collected, the Chief Investigator and the NICTU will provide training to site staff.

Study data will be entered onto a web-based Clinical Trial Database by NICTU personnel and processed electronically. Data quality control checks will be carried out by a data manager to ensure accuracy; data errors will be documented in Quality Control Reports with corrective actions implemented. Data validation will be implemented and discrepancy reports will be generated following data entry to identify discrepancies such as out of range, inconsistencies or protocol deviations based on data validation checks programmed in the clinical trial database.

6. Sample size

The sample size was determined upon the basis of setting a target of the number of reactivated (active) DMO and PDR patients which would enable sensitivity to be tested against a pre-specified target level of 80% (15). This level was considered the minimum acceptable level for the new pathway (ophthalmic grader pathway) to be clinically viable. A lower specificity is considered acceptable and a target of 65% for specificity was used to confirm sufficiency of the sample size for assessing specificity. To detect the sensitivity of the new pathway (photographer/imaging technician pathway) with 80% and 90% power (10% and 12% higher than the 80% minimal target set) would require 89 participants with each DMO/ PDR who have reactivated (active DMO/PDR), with 2-sided 5% significance level (16). Ninety-three participants who have not reactivated would enable a specificity 15% (10%) higher than the 65% target to be detected with 90% power. A 95% confidence interval for photographer sensitivity and specificity would have a confidence interval (Wilson method) with a width of 10-20% depending on the observed level (17). Allowing for 10% missing/indeterminate results, 104 individuals who have re-activated and 104 who have not, are required (208 for each, DMO and PDR) which leads to a need for a maximum of 416 participants in the study overall; some participants may have both DMO and PDR contributing to both DMO and PDR targets.

For peer review only

7. Data Analysis Plan

Outcomes for DMO and PDR will be assessed in two separate analyses. Participants will be categorised as having active/inactive DMO/PDR according to the diagnosis established at the standard care pathway on the person level (i.e. using data from both eyes where appropriate). This reflects the consequences of the clinical decision. The diagnostic performance of the new pathway will be quantified and compared with the standard care pathway. Reflecting how the new pathway would function in practice, an “unsure” classification or an “active” classification will both require an examination by an ophthalmologist. Sensitivity analyses will include assessment of the impact of the “unsure” test classification and of the ophthalmic grader‘s grade upon the diagnostic performance.

The impact of using ultra-wide field images instead of 7 field ETDRS on the diagnostic performance of the new pathway will also be assessed under the principal analyses for PDR detection. In addition, for PDR, a sensitivity analysis will assess

For peer review only - http://bmjopen.bmj.com/site/about/guidelines.xhtml

the diagnostic performance of the ophthalmic grader against the "enhanced" reference standard.

Sensitivity, specificity, positive and negative likelihood ratios will be calculated [with appropriate 95% confidence intervals (CIs)] for the alternative strategy using the current standard of care pathway findings as the reference standard. Agreement (concordance) between the new and current standard of care pathways will also be calculated (with 95% Wilson CI) (17). The difference in sensitivity and specificity between ultra-wide field and 7 field ETDRS images assessed by the ophthalmic graders will be compared with corresponding 95% CIs produced using Newcombe's method for paired data (18).

The proportion of patients requiring subsequent full clinical assessment or unable to undergo assessments, with inadequate quality images or indeterminate findings will be calculated for the alternative pathway with corresponding CIs. All analyses will be carried out using STATA 15 (19).

A detailed Statistical Analysis Plan (SAP) will be written by the trial statistician prior to the final analysis.

8. Health Economic Analysis

This analysis will take into account: 1) sensitivity and specificity of the ophthalmic grader pathway for both DMO and PDR; 2) whether this new pathway detects any PDR missed by the current standard of care; and 3) relative costs.

In the ophthalmic grader pathway, patients will be in one of four groups depending on the decisions made by these staff after reading the images: 1) true negative – no treatment required and patient will return for follow-up at the usual interval; 2) true positive – referred for ophthalmic assessment and treatment as required; 3) false negative – patient who may come to harm by visual loss; 4) false positive – patient will be referred to the ophthalmologist but will not require treatment; these patients will not come to harm apart from possible anxiety and inconvenience, but will consume ophthalmologist time.

Scores obtained in the health-related quality of life questionnaire (EQ-5D-5L) and visual function questionnaires (NEI VFQ-25 and Vis-QoL) will provide utility data for different health states. Resource use data will be collected to explore the costs of delivering the standard care pathway and the ophthalmic grader pathway and to find the key cost drivers. This will mainly consist of staff costs. Costs of 7-field ETDRS images will be compared with those of ultra-wide angle imaging.

If the sensitivity and specificity of the new and standard pathways were exactly the same there would be no quality-adjusted life year (QALY) differences, though there might be some disutility from process changes (i.e. if one pathway caused more anxiety than the other). The key gain would be ophthalmologist time freed for other activities. The real benefits might be reduction in waiting times and earlier treatment of other patients leading to QALY gains for them. Such benefits would be difficult to estimate and the simplest measure would be ophthalmologist sessions/days released for other activities. However, we will identify ways in which time released would be used. An underlying assumption to be checked is that the cost of assessment by the ophthalmic graders is less than that of the ophthalmologists. Both pathways would require nurse or optometrist time for checking visual acuity, as done in routine clinical practice. The costs of the image-based pathways will include both time for taking and reading images, using both

For peer review only - http://bmjopen.bmj.com/site/about/guidelines.xhtml

conventional and ultra-wide field cameras. If there was marginal loss in sensitivity from the new pathway the consequences could be visual loss before next visit was due, or detection at next visit (with or without visual loss occurring) followed by possibly later than optimal treatment. Both could have disutilities.

It should be noted that assessment in clinical practice is repeated over time so active disease missed at one assessment might be picked up at the next. Given the cross-sectional design of EMERALD and the fact that all patients will undergo the standard care pathway, we will not be able to assess the disutility of any visual changes in patients recruited.

We will develop a Markov model using data from both this study (the EQ-5D 5L data for different states) and published studies on progression, so analysis of the effect of a reduced sensitivity will include, for DMO and PDR separately: 1) probability of progression before next visit, 2) probability that this would lead to irreversible visual loss and if so, how much; 3) if there was irreversible visual loss, the resulting disutility and QALY loss.

Specificity would be the determining factor in savings in ophthalmologist time: the poorer the specificity the lower the savings. In the PDR group, ultra-wide field imaging will be compared with both, standard care (ophthalmologist evaluating patients by slit-lamp biomicroscopy) and 7-field ETDRS to assess the cost-effectiveness of this more recently introduced imaging modality.

Any future costs and benefits will be discounted at 3.5%. NHS and personal social services perspective will be adopted. The model will be populated by cost, sensitivity and specificity data from the study and by estimates of progression, effectiveness of treatment (prompt and delayed), quality of life and future costs from published literature and expert opinion. Results will be expressed as cost per QALY gained. Appropriate sensitivity analyses will be conducted to assess robustness of results. Probabilistic sensitivity analyses will be undertaken to explore uncertainty in model parameters and to allow presentation of cost-effectiveness acceptability curves.

9. Focus Groups Discussions: Assessment of Acceptability of the New Care Pathway

The acceptability of the ophthalmic graders pathway will be evaluated through focus group discussions. Patients' views on the acceptability of the proposed new pathway are essential if this were to be incorporated into clinical practice. Focus group discussions are particularly useful to help identify issues that resonate with lay people in matters of health care and have been widely used in health services research; focus group discussions reach the parts that other methods cannot (20). Their use in this study will enable us to acquire data on a full range of issues – some of which are unlikely to have been anticipated by professionals. The sample frame is designed to include consenting participants drawn from different UK areas and different age groups. Group discussions will be facilitated by a trained researcher. Discussions will be audio-recorded and later transcribed for analysis.

Informed consent will be obtained from participants prior to the focus group discussions. Participants consenting to take part will be approached at a later date via letter/phone call to inform them about the date/location/time of the meeting.

EMERALD will also examine the acceptability of the new pathway to health professionals. For this purpose, a small number of focus groups will be conducted

For peer review only - http://bmjopen.bmj.com/site/about/guidelines.xhtml

involving photographer/imaging technicians/graders and ophthalmologists, all recruited from staff at participating sites.

The data from the focus groups will be analysed by the use of simple content analysis strategies. The focus of the analysis will be on ‘acceptability’ of the new alternative pathway and factors that might facilitate or impede such acceptability (21).

Ethics and Dissemination

Ethics approval was obtained from the Office for Research Ethics Committees Northern Ireland (ORECNI - 17/NI/0124).

The study is conducted in compliance with the protocol approved by the Research Ethics Committee (REC). Changes to the protocol may require REC approval prior to implementation. The NICTU in collaboration with the sponsor will submit all protocol modifications to the REC for review in accordance with the governing regulations.

Serious Adverse Events (SAE) will be recorded and reported to ORECNI.

In order to maintain participant’s confidentiality, a unique study identification number will be used for each participant. Study reports and communication regarding the study will identify the patients by their assigned unique trial identifier. Computers where information will be stored will be password protected.

A report containing the methodology and results of EMERALD will be published as a Health Technology Assessment monograph, freely accessible via the NIHR HTA web site. The Royal College of Ophthalmologist will be contacted when the study is completed to allow the study findings to be incorporated in future Diabetic Retinopathy guidelines. Findings will be published in national/international peer reviewed journals and presented at national/international meetings and patient groups.

References

[1] Liew G, Michaelides M, Bunce C. A comparison of the causes of blindness certifications in England and Wales in working age adults (16-64 years), 1999-2000 with 2009-2010. *BMJ Open* 2014, 4:e004015.

[2] Zheng Y, He M, Congdon N. The worldwide epidemic of diabetic retinopathy. *Indian J Ophthalmol* , 2012;60:428-31.

[3] Minassian DC, Owens DR, Reidy A. Prevalence of diabetic macular oedema and related health and social care resource use in England. *Br J Ophthalmol*. 2012;96:345-9.

[4] Yau JW, Rogers SL, Kawasaki R, et al: Meta-Analysis for Eye Disease (META-EYE) Study Group. Global prevalence and major risk factors of diabetic retinopathy. *Diabetes Care*, 2012;35:556-64.

For peer review only - http://bmjopen.bmj.com/site/about/guidelines.xhtml

[5] Keenan TD, Johnston RL, Donachie PH et al. United Kingdom National Ophthalmology Database Study: Diabetic Retinopathy; Report 1: prevalence of centre-involving diabetic macular oedema and other grades of maculopathy and retinopathy in hospital eye services. *Eye* , 2013;27:1397-404.

[6] Diabetic Retinopathy Clinical Research Network, Elman MJ, Aiello LP, Beck RW, et al. Randomized trial evaluating ranibizumab plus prompt or deferred laser or triamcinolone plus prompt laser for diabetic macular edema. *Ophthalmology* 2010;117:1064- 1077.

[7] Mitchell P, Bandello F, Schmidt-Erfurth U,et al. The RESTORE study: ranibizumab monotherapy or combined with laser versus laser monotherapy for diabetic macular edema. *Ophthalmology* 2011;118:615-25.

[8] Nguyen QD, Brown DM, Marcus DM et al. Ranibizumab for diabetic macular edema: results from 2 phase III randomized trials: RISE and RIDE. *Ophthalmology* 2012;119:789-801.

[9] Korobelnik JF, Do DV, Schmidt-Erfurth U, et al. Intravitreal aflibercept for diabetic macular edema. *Ophthalmology* 2014;121:2247-2254.

[10] Diabetic Retinopathy Clinical Research Network, Wells JA, Glassman AR, Ayala AR, et al. Aflibercept, bevacizumab, or ranibizumab for diabetic macular edema. *New Engl J Med* 2015;372:1193-203.

[11] Virgili G, Menchini F, Casazza G, et al. Optical coherence tomography (OCT) for detection of macular oedema in patients with diabetic retinopathy. *Cochrane Database Syst Rev.* 2015;1:CD008081.

[12] Olson J, Sharp P, Goatman K, et al. Improving the economic value of photographic screening for optical coherence tomography-detectable macular oedema: a prospective, multicentre, UK study. *Health Technol Assess*. 2013;17:1-142.

[13] Knottnerus JA, Muris JW. Assessment of the accuracy of diagnostic tests: the cross-sectional study. *Journal of Clinical Epidemiology* 2003;56:1118–1128.

[14] Whiting PF, Rutjes AWS, Westwood ME, et al. QUADAS-2: A Revised Tool for the Quality Assessment of Diagnostic Accuracy Studies. *Ann Intern Med* 2011;155:529-536.

[15] Obuchowski NA. Sample size calculations in studies of test accuracy. *Statistical Methods in Medical Research* 1998;7:371-392.

[16] Silva PS, Cavallerano JD, Tolson AM, et al. Real-Time Ultrawide Field Image Evaluation of Retinopathy in a Diabetes Telemedicine Program. *Diabetes Care*, 2015;38:1643-9.

[17] Piegorsch WW. Sample sizes for improved binomial confidence intervals. *Computational Statistics & Data Analysis* 2004;46:309 – 316.

For peer review only - http://bmjopen.bmj.com/site/about/guidelines.xhtml

[18] Newcombe RG. Improved confidence intervals for the difference between binomial proportions based on paired data. *Stats in Medicine* 1998;17:2635-2650.

[19] StataCorp. 2017. Stata Statistical Software: Release 14. College Station, TX: StataCorp LP.

[20] Kitzinger, J. (1995). Qualitative Research: Introducing focus groups, *BMJ*;311:299-302.

[21] Prior, L. Content Analysis. In, P. Leavy. Ed. *The Oxford Handbook of Qualitative Research Methods*. 2014; Oxford Library.

For peer review only

Legends for Figures

Figure 1: Ophthalmic Grader's Pathway, Summary.

Figure 2: Study Flowchart.

For peer review only

For peer review only - http://bmjopen.bmj.com/site/about/guidelines.xhtml

Authorship policy: An author is considered someone who has made a substantive intellectual contribution to the study and the relevant report.

EMERALD STUDY GROUP: Ahmed Saad, James Cook University Hospital, South Tees Hospitals NHS Foundation Trust; Andrew Jackson, Northern Ireland Clinical Trials Unit; Augusto Azuara-Blanco, Queen's University and Royal Victoria Hospital, Belfast H&SC Trust; Caroline Styles, Queen's Margaret Hospital, Fife; Christine McNally, Northern Ireland Clinical Trials Unit; Clare Bailey, Bristol Eye Hospital, University Hospitals Bristol NHS Foundation Trust; Danny McAuley, Queen's University and Royal Victoria Hospital, Belfast H&SC Trust; David H Steel, Sunderland Eye Infirmary, City Hospitals Sunderland NHS Foundation Trust; Faruque Ghanchi, Bradford Teaching Hospitals NHS Trust; Geeta Menon, Frimley Park Hospital NHS Foundation Trust; Haralabos Eleftheriadis, Kings College Hospital NHS Foundation Trust; Hema Mistry, Warwick University; Jonathan Cook and William Sones, Centre for Statistics in Medicine, University of Oxford; Lindsay Prior, Centre for Public Health, Queens University, Belfast; Nachiketa Acharya, Sheffield Teaching Hospitals NHS Foundation Trust; Noemi Lois, Queen's University and Royal Victoria Hospital Belfast H&SC Trust; Norman Waugh, Warwick University; Rachael Rice, Northern Ireland Clinical Trials Unit; Samia Fatum, John Radcliffe Hospital, Oxford University Hospitals NHS Foundation Trust; Sobha Sivaprasad, Moorfields Eye Hospital NHS Foundation Trust; Stephen Aldington and Peter Scanlon, Gloucestershire Hospitals NHS Foundation Trust; Tariq M Aslam, Manchester Royal Eye Hospital, Central Manchester University Hospitals NHS Foundation Trust; Victor Chong, Royal Free Hospital NHS Foundation Trust, London.

Authors' Contributions: NL conceived the study, drafted and refined the protocol in conjunction with the collaborators. JC and NL determined the sample size for the study and statistical analysis plan. NW and HM planned the cost-effectiveness evaluation. LP designed and planned the focus group discussions and will carry them out. All authors provided substantial contributions to the design of the work, drafted and approved the final manuscript submission and are in agreement to be accountable for all aspects of the work.

Clinical sites participating in recruitment: Bradford Royal Infirmary, Bradford Teaching Hospitals NHS Foundation Trust; Bristol Eye Hospital, University Hospitals Bristol NHS Foundation Trust; Frimley Park Hospital NHS Foundation Trust; Gloucestershire Royal Hospital, Gloucestershire Hospitals NHSF Trust; James Cook University Hospital, South Tees Hospitals NHS Foundation Trust;Kings College Hospital NHS Foundation Trust; Manchester Royal Eye Hospital, Central Manchester University Hospitals NHS Foundation Trust; Moorfields Eye Hospital NHS Foundation Trust; John Radcliffe Hospital, Oxford University Hospitals NHS Foundation Trust; Queen Margaret Hospital, Fife; Royal Victoria Hospital, Belfast H&SC Trust; Sheffield Teaching Hospitals NHS Foundation Trust; Sunderland Eye Infirmary, City Hospitals Sunderland NHS Foundation Trust.

EMERALD is currently recruiting. The first trial participant was recruited in October 2017.

For peer review only - http://bmjopen.bmj.com/site/about/guidelines.xhtml

Acknowledgement: To optometrists, research coordinators, research nurses, ophthalmic technicians and ophthalmic photographers contributing to EMERALD at each of the participating sites. To all patients participating in EMERALD. To Alison Murphy and Lynn Murphy and to Nuala Hannaway for their help and assistance with this clinical trial.

Trial Management Group: Prof Noemi Lois (Chief Investigator), Prof. Augusto Azuara-Blanco, Mr Steve Aldington, Dr Danny McAuley, Mr Peter Scanlon, Mr Lindsay Prior, Mrs Clare Newall, Mrs Michelle McGaughey, Mrs Christine McNally (Trial Manager), Miss Rachael Rice (Clinical Trial Co-ordinator), Mr Andrew Jackson (Data Manager), Mr Jonathan Cook (Statistician), Mr William Sones (Statistician) Mr Norman Waugh (Health Economist), Ms Hema Mistry (Health Economist), Mr Mark Wilson (Database Developer), Mrs Nuala Hannaway (Clinical Trial Administrator), Mrs Catherine Campbell (Clinical Research Monitor).

Trial Steering Committee: Mr John Norrie (Chair); Mr David Owens; Mrs Florence Findlay-White; Mr Jonathan Cook; Mr William Sones; Mr Winfried Amoaku; Miss Yemisi Takwoingi.

The roles and responsibilities of the TSC can be found in https://www.nihr.ac.uk/funding-and-support/documents/funding-for-research-studies/how-to-apply/NETSCC_Project_Oversight_Groups_Guidance.pdf

A study Data and Ethics Monitoring Committee was not deemed necessary given the extremely low risk of this diagnostic study.

Trial Sponsor: The Belfast Health and Social Care Trust. Belfast, NI. Contact person: Ms Alison Murphy; alison.murphy@belfasttrust.hscni.net.

Contact for public queries: EMERALD@nictu.hscni.net

Funding Statement

This project was funded by the NIHR [Health Technology Assessment programme] (project number 13/142/04).

The views expressed are those of the authors and not necessarily those of the NHS, the NIHR or the Department of Health and Social Care.

Neither the Sponsor nor the Funder had any role on the study design; collection, management, analysis, and interpretation of data; writing of this manuscript or in the decision to submit this manuscript for publication.

Competing Interests Statement

Noemi Lois: None; Norman Waugh: None; Augusto Azuara-Blanco: None; Hema Mistry: None; Danny McAuley: None; Tariq Aslam: None; Clare Bailey: has been ad-hoc advisor for Alcon, Bayer, Novartis, Alimera sciences and Allergan; Victor Chong

For peer review only - http://bmjopen.bmj.com/site/about/guidelines.xhtml

is a part time employee of Boehringer Ingelheim International GmBH (BII), Germany; this study, however, is not being undertaken as part of the employment with BII and, thus, the content of this manuscript is not endorsed by BII. Victor Chong has received also speaker fees from Quantel Medical, France; Sobha Sivaprasad has received research grants, travel feeds and attended advisory board meetings of Novartis, Bayer, Roche, Allergan, Heidelberg Engineering, Optos Plc an Boehringer Ingleheim; David Steel; David Steel acted as Consultant to Alcon, attended advisory boards for Novartis and Bayer and received research funding from Bayer and Alcon. Peter Scanlon has attended Advisory Boards for Allergan, Roche, Boehringer and Bayer; his department has received Educational, Research or Audit Grants from Allergan, Novartis and Bayer.

Data Access

The Chief investigator has access to the trial data and will act as a data guardian.

Word Count: 4477, excluding abstract & references.

For peer review only

Ophthalmic Grader Pathway

- Images will be obtained from both eyes by an ophthalmic photographer/imaging technician, including:
  - Spectral Domain Optical Coherence Tomography (SD-OCT)
  - Early Treatment Diabetic Retinopathy Study (ETDRS) 7 field non-stereoscopic fundus photographs
  - Ultra-wide angle fundus images
- Images will be read by trained ophthalmic graders
  - Presence/absence of active DMO/PDR determined

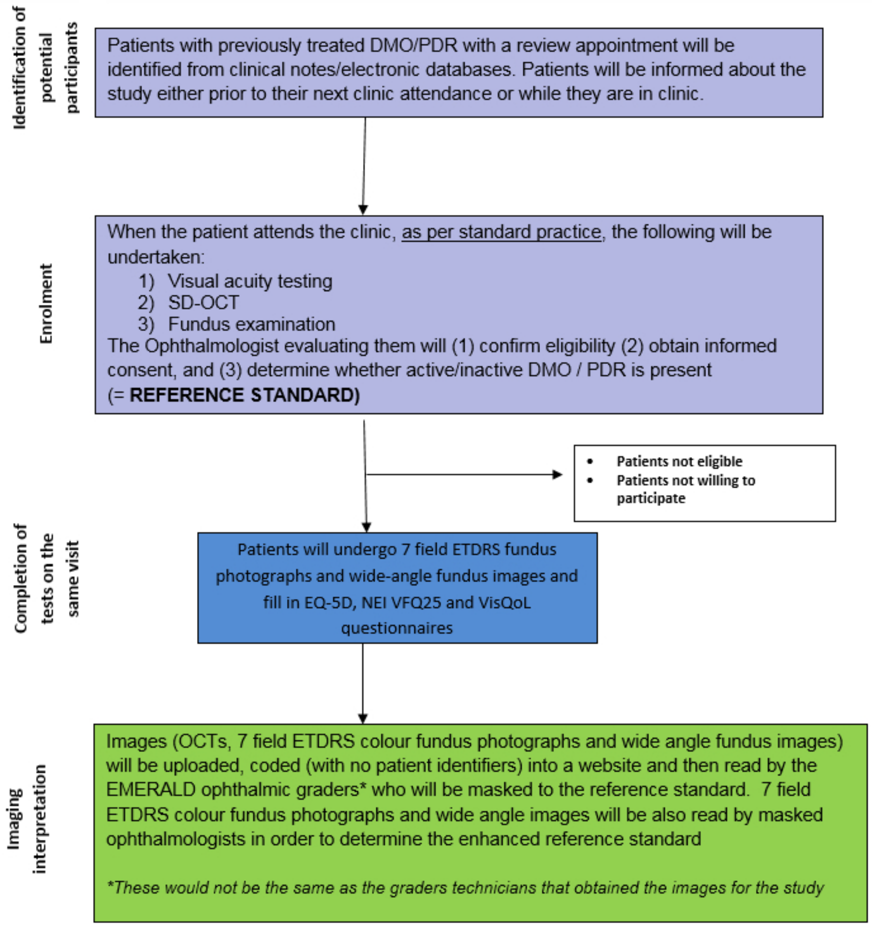

DMO= Diabetic Macular Oedema; PDR=Proliferative Diabetic Retinopathy; SD-OCT=Spectral Domain Optical Coherence Tomography; ETDRS=Early Treatment Diabetic Retinopathy Study

Reporting checklist for protocol of a clinical trial.

Based on the SPIRIT guidelines.

Instructions to authors

Complete this checklist by entering the page numbers from your manuscript where readers will find each of the items listed below.

Your article may not currently address all the items on the checklist. Please modify your text to include the missing information. If you are certain that an item does not apply, please write "n/a" and provide a short explanation.

Upload your completed checklist as an extra file when you submit to a journal.

In your methods section, say that you used the SPIRIT reporting guidelines, and cite them as:

Chan A-W, Tetzlaff JM, Altman DG, Laupacis A, Gøtzsche PC, Krleža-Jerić K, Hróbjartsson A, Mann H, Dickersin K, Berlin J, Doré C, Parulekar W, Summerskill W, Groves T, Schulz K, Sox H, Rockhold FW, Rennie D, Moher D. SPIRIT 2013 Statement: Defining standard protocol items for clinical trials. Ann Intern Med. 2013;158(3):200-207

		Reporting Item	Page Number
Title	#1	Descriptive title identifying the study design, population, interventions, and, if applicable, trial acronym	1
Trial registration	#2a	Trial identifier and registry name. If not yet registered, name of intended registry	1

For peer review only

Trial registration: data set	#2b	All items from the World Health Organization Trial Registration Data Set	1
Protocol version	#3	Date and version identifier	1
Funding	#4	Sources and types of financial, material, and other support	17
Roles and responsibilities: contributorship	#5a	Names, affiliations, and roles of protocol contributors	1, 16
Roles and responsibilities: sponsor contact information	#5b	Name and contact information for the trial sponsor	17
Roles and responsibilities: sponsor and funder	#5c	Role of study sponsor and funders, if any, in study design; collection, management, analysis, and interpretation of data; writing of the report; and the decision to submit the report for publication, including whether they will have ultimate authority over any of these activities	17
Roles and responsibilities: committees	#5d	Composition, roles, and responsibilities of the coordinating centre, steering committee, endpoint adjudication committee, data management team, and other individuals or groups overseeing the trial, if applicable (see Item 21a for data monitoring committee)	17

For peer review only

Background and rationale	#6a	Description of research question and justification for undertaking the trial, including summary of relevant studies (published and unpublished) examining benefits and harms for each intervention	3,4
Background and rationale: choice of comparators	#6b	Explanation for choice of comparators	3,4
Objectives	#7	Specific objectives or hypotheses	3,4
Trial design	#8	Description of trial design including type of trial (eg, parallel group, crossover, factorial, single group), allocation ratio, and framework (eg, superiority, equivalence, non-inferiority, exploratory)	4,5
Study setting	#9	Description of study settings (eg, community clinic, academic hospital) and list of countries where data will be collected. Reference to where list of study sites can be obtained	5
Eligibility criteria	#10	Inclusion and exclusion criteria for participants. If applicable, eligibility criteria for study centres and individuals who will perform the interventions (eg, surgeons, psychotherapists)	5
Interventions: description	#11a	Interventions for each group with sufficient detail to allow replication, including how and when they will be administered	6,7,8

For peer review only

Interventions: modifications	#11b	Criteria for discontinuing or modifying allocated interventions for a given trial participant (eg, drug dose change in response to harms, participant request, or improving / worsening disease)	N/A
Interventions: adherance	#11c	Strategies to improve adherence to intervention protocols, and any procedures for monitoring adherence (eg, drug tablet return; laboratory tests)	N/A
Interventions: concomitant care	#11d	Relevant concomitant care and interventions that are permitted or prohibited during the trial	N/A
Outcomes	#12	Primary, secondary, and other outcomes, including the specific measurement variable (eg, systolic blood pressure), analysis metric (eg, change from baseline, final value, time to event), method of aggregation (eg, median, proportion), and time point for each outcome. Explanation of the clinical relevance of chosen efficacy and harm outcomes is strongly recommended	5,6
Participant timeline	#13	Time schedule of enrolment, interventions (including any run-ins and washouts), assessments, and visits for participants. A schematic diagram is highly recommended (see Figure)	Figures 1,2
Sample size	#14	Estimated number of participants needed to achieve study objectives and how it was determined, including clinical and statistical assumptions supporting any sample size calculations	10

For peer review only

Recruitment	#15	Strategies for achieving adequate participant enrolment to reach target sample size	Figure 2
Allocation: sequence generation	#16a	Method of generating the allocation sequence (eg, computer-generated random numbers), and list of any factors for stratification. To reduce predictability of a random sequence, details of any planned restriction (eg, blocking) should be provided in a separate document that is unavailable to those who enrol participants or assign interventions	N/A
Allocation concealment mechanism	#16b	Mechanism of implementing the allocation sequence (eg, central telephone; sequentially numbered, opaque, sealed envelopes), describing any steps to conceal the sequence until interventions are assigned	N/A
Allocation: implementation	#16c	Who will generate the allocation sequence, who will enrol participants, and who will assign participants to interventions	N/A
Blinding (masking)	#17a	Who will be blinded after assignment to interventions (eg, trial participants, care providers, outcome assessors, data analysts), and how	9,10
Blinding (masking): emergency unblinding	#17b	If blinded, circumstances under which unblinding is permissible, and procedure for revealing a participant's allocated intervention during the trial	N/A

For peer review only

Data collection plan	#18a	Plans for assessment and collection of outcome, baseline, and other trial data, including any related processes to promote data quality (eg, duplicate measurements, training of assessors) and a description of study instruments (eg, questionnaires, laboratory tests) along with their reliability and validity, if known. Reference to where data collection forms can be found, if not in the protocol	8,9,10
Data collection plan: retention	#18b	Plans to promote participant retention and complete follow-up, including list of any outcome data to be collected for participants who discontinue or deviate from intervention protocols	N/A
Data management	#19	Plans for data entry, coding, security, and storage, including any related processes to promote data quality (eg, double data entry; range checks for data values). Reference to where details of data management procedures can be found, if not in the protocol	10
Statistics: outcomes	#20a	Statistical methods for analysing primary and secondary outcomes. Reference to where other details of the statistical analysis plan can be found, if not in the protocol	11,12,13
Statistics: additional analyses	#20b	Methods for any additional analyses (eg, subgroup and adjusted analyses)	11,12,13

For peer review only

Statistics: analysis population and missing data	#20c	Definition of analysis population relating to protocol non-adherence (eg, as randomised analysis), and any statistical methods to handle missing data (eg, multiple imputation)	11,12,13
Data monitoring: formal committee	#21a	Composition of data monitoring committee (DMC); summary of its role and reporting structure; statement of whether it is independent from the sponsor and competing interests; and reference to where further details about its charter can be found, if not in the protocol. Alternatively, an explanation of why a DMC is not needed	17
Data monitoring: interim analysis	#21b	Description of any interim analyses and stopping guidelines, including who will have access to these interim results and make the final decision to terminate the trial	N/A
Harms	#22	Plans for collecting, assessing, reporting, and managing solicited and spontaneously reported adverse events and other unintended effects of trial interventions or trial conduct	N/A
Auditing	#23	Frequency and procedures for auditing trial conduct, if any, and whether the process will be independent from investigators and the sponsor	N/A
Research ethics approval	#24	Plans for seeking research ethics committee / institutional review board (REC / IRB) approval	13

For peer review only

Protocol amendments	#25	Plans for communicating important protocol modifications (eg, changes to eligibility criteria, outcomes, analyses) to relevant parties (eg, investigators, REC / IRBs, trial participants, trial registries, journals, regulators)	13
Consent or assent	#26a	Who will obtain informed consent or assent from potential trial participants or authorised surrogates, and how (see Item 32)	8
Consent or assent: ancillary studies	#26b	Additional consent provisions for collection and use of participant data and biological specimens in ancillary studies, if applicable	N/A
Confidentiality	#27	How personal information about potential and enrolled participants will be collected, shared, and maintained in order to protect confidentiality before, during, and after the trial	10,13
Declaration of interests	#28	Financial and other competing interests for principal investigators for the overall trial and each study site	17,18
Data access	#29	Statement of who will have access to the final trial dataset, and disclosure of contractual agreements that limit such access for investigators	18
Ancillary and post trial care	#30	Provisions, if any, for ancillary and post-trial care, and for compensation to those who suffer harm from trial participation	N/A

For peer review only

Dissemination policy: trial results	#31a	Plans for investigators and sponsor to communicate trial results to participants, healthcare professionals, the public, and other relevant groups (eg, via publication, reporting in results databases, or other data sharing arrangements), including any publication restrictions	13
Dissemination policy: authorship	#31b	Authorship eligibility guidelines and any intended use of professional writers	16
Dissemination policy: reproducible research	#31c	Plans, if any, for granting public access to the full protocol, participant-level dataset, and statistical code	N/A
Informed consent materials	#32	Model consent form and other related documentation given to participants and authorised surrogates	1
Biological specimens	#33	Plans for collection, laboratory evaluation, and storage of biological specimens for genetic or molecular analysis in the current trial and for future use in ancillary studies, if applicable	N/A

Author notes

The SPIRIT checklist is distributed under the terms of the Creative Commons Attribution License CC-BY-ND 3.0. This checklist was completed on 05. September 2018 using http://www.goodreports.org/, a tool made by the EQUATOR Network in collaboration with Penelope.ai

For peer review only

BMJ Open

Effectiveness of Multimodal imaging for the Evaluation of Retinal oedema And new vesseLs in Diabetic retinopathy (EMERALD): Protocol for a Diagnostic Study

Journal:	*BMJ Open*
Manuscript ID	bmjopen-2018-027795.R1
Article Type:	Protocol
Date Submitted by the Author:	16-Apr-2019
Complete List of Authors:	Lois, Noemi; Wellcome-Wolfson Institute for Experimental Medicine, Ophthalmology<br>Cook, Jonathan; University of Oxford, NDORMS<br>Aldington, Stephen; Gloucester Hospitals NHS Foundation Trust<br>Waugh, Norman; University of Warwick, Warwick Evidence<br>Mistry, Hema; University of Warwick, Warwick Evidence<br>Sones, William; University of Oxford, Centre for Statistic in Medine, Oxford, UK OX37LD 01865223450<br>McAuley, Danny; Queen's University Belfast, Centre for Infection and Immunity<br>Aslam, Tariq; University of Manchester,<br>Bailey, Claire; University of Bristol<br>Chong, Victor; Royal Free Hospital NHS Foundation Trust, Ophthalmology<br>Ghanchi, Faruque; Bradford Royal Infirmary, Ophthalmology<br>Scanlon, Peter; Gloucestershire Hospitals NHS Foundation Trust<br>Sivaprasad, Sobha; Consultant Ophthalmologist, Laser and Retinal Research Unit<br>Steel, David; Sunderland Eye Infirmary; Institute of Genetic Medicine, Newcastle University<br>Styles, Caroline; Queen Margaret Hospital, Ophthalmology<br>McNally, Christine; Northern Ireland Clinical Trials Unit<br>Rice, Rachael ; Northern Ireland Clinical Trials Unit<br>Prior, Lindsay; Queens University Belfast<br>Azuara-Blanco, Augusto ; Queens University Belfast , Wellcome-Wolfson Institute for Experimental Medicine
**Primary Subject Heading**:	Ophthalmology
Secondary Subject Heading:	Diabetes and endocrinology, Diagnostics, Evidence based practice, Health economics, Health services research
Keywords:	Diabetic retinopathy < DIABETES & ENDOCRINOLOGY, HEALTH ECONOMICS, Medical retina < OPHTHALMOLOGY, PUBLIC HEALTH, QUALITATIVE RESEARCH, Organisational development < HEALTH SERVICES ADMINISTRATION & MANAGEMENT

SCHOLARONE™
Manuscripts

Effectiveness of **M**ultimodal imaging for the **E**valuation of **R**etinal oedema **A**nd new vesse**L**s in **D**iabetic retinopathy (EMERALD): Protocol for a Diagnostic Study

Noemi Lois, MD[1], PhD, FRCS(Ed), FRCOphthal
Jonathan A Cook, PhD, BSc Hons[2]
Stephen Aldington, PGDip, HND[3]
Norman Waugh, MRCP(UK), MPH[4]
Hema Mistry PhD[4]
William Sones[2]
Danny McAuley MD, FRCP[1, 5]
Tariq M Aslam, DM (Oxon), FRCSEd, PhD[6]
Claire Bailey, MD, BM BCh, MA[7]
Victor Chong, MD, MPHIL, MB ChB[8]
Faruque DaudBhai Ghanchi, MBBS[9]
Peter H Scanlon, MD, FRCP, FRCOphth[3]
Sobha Sivaprasad, DM, FRCS, FRCOphth[10]
David H Steel, MBBS, MD, FRCOphth[11]
Caroline Styles, MB ChB[12]
Christine McNally[13]
Rachael Rice[13]
Lindsay Prior, PhD[14]
Augusto Azuara-Blanco, MD, PhD, FRCS(Ed), FRCOphthal[14]
On behalf of the EMERALD Study Group

From The Wellcome-Wolfson Institute for Experimental Medicine,[1] Queen's University, Belfast; Centre for Statistics in Medicine, University of Oxford;[2] Gloucestershire Hospitals NHS Foundation Trust; [3] The Division of Health Sciences, University of Warwick;[4] The Regional Intensive Care Unit, Royal Victoria Hospital, Belfast;[5] The Manchester Academic Health Science Centre, Manchester Royal Eye Hospital and Division of Pharmacy and Optometry, School of Health Sciences, Faculty of Biology, Medicine and Health, University of Manchester, Manchester;[6] Bristol Eye Hospital, Bristol;[7] Royal Free Hospital NHS Foundation Trust, London;[8] Bradford Teaching Hospitals, Bradford Royal Infirmary, Bradford;[9] NIHR Moorfields Biomedical Research Centre, London;[10] Sunderland Eye Infirmary, Sunderland and Institute of Genetic Medicine, Newcastle University, Newcastle Upon Tyne;[11] Queen Margaret Hospital, Fife, Scotland;[12] The Northern Ireland Clinical Trials Unit (NICTU), Belfast;[13] and the Centre for Public Health, Queens University, Belfast.[14]

Correspondence: Prof. Noemi Lois, Wellcome-Wolfson Institute for Experimental Medicine, Queen's University Belfast, 97 Lisburn Road, BT9 7BL Belfast, UK, n.lois@qub.ac.uk, Telephone: 00 44 28 9097 6462

Trial Registration: ISRCTN Number: 10856638 (registered on 8/03/2017)

Protocol Version: 3 (March 20, 2018). The full protocol, study guidelines and patient related materials can be accessed at the EMERALD website (http://www.nictu.hscni.net/emerald-trial-/)

Key words: Diabetes, Diabetic Macular Oedema, Diabetic Macular Edema, DMO, PDR, Pathway, laser, anti-VEGFs, cost-effectiveness.

This protocol was prepared following SPIRIT and STARD guidelines; all items listed in the World Health Organisation Trial Registration Data Set have been specified throughout this protocol.

For peer review only - http://bmjopen.bmj.com/site/about/guidelines.xhtml

ABSTRACT (250 words)

Introduction
Diabetic macular oedema (DMO) and proliferative diabetic retinopathy (PDR) are the major causes of sight loss in people with diabetes. Due to the increased prevalence of diabetes, the workload related to these complications is increasing making it difficult for Hospital Eye Services (HSE) to meet demands.

Methods and analysis
EMERALD is a prospective, case-referent, cross-sectional diagnostic study which aims at determining the diagnostic performance, cost-effectiveness and acceptability of a new form of surveillance for people with stable DMO and/or PDR which entails multimodal imaging and image review by an ophthalmic grader, using the current standard of care (evaluation of patients in clinic by an ophthalmologist) as the reference standard. If safe, cost-effective and acceptable, this pathway could help HES by freeing ophthalmologist time. The primary outcome of EMERALD is sensitivity of the new surveillance pathway in detecting active DMO/PDR. Secondary outcomes include specificity, agreement between new and the standard care pathway, positive and negative likelihood ratios, cost-effectiveness, acceptability, proportion of patients requiring subsequent full clinical assessment, unable to undergo imaging, with inadequate quality images or indeterminate findings.

Ethics and dissemination
Ethical approval was obtained for this study from the Office for Research Ethics Committees Northern Ireland (reference 17/NI/0124). Study results will be published as a Health Technology Assessment monograph, in peer-reviewed national and international journals and presented at national/international conferences and to patient groups.

Registration details
ISRCTN: 10856638 (date of registration 08/03/2017)

For peer review only

Strengths and limitations of study

Strengths:
- Prospective, multicenter study
- Masking of graders to results of the reference standard and masking of ophthalmologists to results of the grader's pathway.
- Evaluation of the acceptability of the new pathway to patients and health professionals
- Early involvement in all aspects of the study of patients and public

Limitations:
- Lack of fluorescein angiography to confirm active proliferative diabetic retinopathy.

Introduction

Diabetic Retinopathy (DR) is the most common microvascular complication of diabetes and a leading cause of visual loss among individuals of working age (1). Patients with DR may lose sight as a result of the development of diabetic macular oedema (DMO) and/or proliferative diabetic retinopathy (PDR), the major complications of DR. In the former, fluid accumulates in the central part of the retina, the macula, which is responsible for detailed central vision. In the latter, abnormal new blood vessels ("new vessels") grow on the optic nerve head or on the surface of the retina and towards the inside of the eye (the vitreous cavity) leading to sight loss from haemorrhaging or/and traction on the retina with subsequent retinal detachment. Due to the increasing numbers of people with diabetes, it is expected that the burden of DR will continue to rise. Indeed, it has been estimated that the worldwide prevalence of DR will increase from 126.6 million in 2010 to 191 million by 2030 (2).

The prevalence of DMO in England was estimated to be 7% of the total diabetic population in 2010 (3). A very similar estimate of the prevalence was found in an individual participant data meta-analysis, which included 22,896 individuals from 35 studies, conducted in Asia, Australia, Europe, and US, which provided an overall age-standardised prevalence of DMO of 6.8% (4). Based on this published prevalence of DMO and considering the prevalence of diabetes in the UK (5) it can be estimated that there are 280,000 people in the UK affected by DMO. In the UK, patients with DMO are treated with focal or grid macular laser [when the central retinal thickness (CRT), measured by means of spectral domain optical coherence tomography (SD-OCT), is <400 microns], which is delivered in a single session, or with injections into the eye of anti-vascular endothelial growth factor (anti-VEGF) therapies, currently in the National Health Service (NHS) ranibizumab (Lucentis) and aflibercept (Eylea) [when the central retinal thickness measured by SD-OCT is ≥400 microns]. (6, 7) Intraocular steroids are also available for patients that do not respond to the above therapies and are pseudophakic (i.e. have had their cataracts removed) (8). Once treated, long-term follow-up is required, to determine whether DMO recurs. Typically, patients are followed every three to four months following laser treatment for DMO or monthly initially and then every 1-3 months thereafter following treatment with anti-VEGFs [9]. Follow-up continues for the rest of the patient's life.

The estimated prevalence of PDR in the individual participant data meta-analysis referred to above (4) was 6.96%; based on the prevalence of diabetes in UK [5] there are ~278,400 people in the UK affected by PDR. At present in the UK, patients with PDR are treated with panretinal laser photocoagulation (PRP), which is delivered most often in two to three sessions. Once treatment is completed, patients are followed at 4-6 month intervals for their lifetime to determine whether reactivation occurs, as new vessels in PDR could come back and tractional retinal detachment can still ensue (9). A high proportion of patients with DR followed in Hospital Eye Services (HES) have treated and inactive PDR (10).

Currently in the NHS ophthalmologists with expertise in retinal diseases assess patients during follow up visits. At each visit, patients with DMO are evaluated with a visual acuity test, most often undertaken by a nurse; SD-OCT, obtained by a photographer and interpreted by the ophthalmologist, and slit-lamp biomicroscopy, undertaken by an ophthalmologist, who then determines whether DMO is present or absent. SD-OCT is a non-invasive, user-friendly and safe imaging technique that obtains scans of the back of the eye. SD-OCT allows measurement of the CRT (which is often increased when DMO is present) and visualising fluid in

For peer review only - http://bmjopen.bmj.com/site/about/guidelines.xhtml

the retina which is the hallmark of DMO. SD-OCT has been extensively used in clinical trials and clinical practice to determine the presence of DMO, select treatment, and monitor the response to treatment (11-17). In the follow-up of patients with PDR ophthalmologists typically examine the patient by slit-lamp biomicroscopy. Photographs are not routinely obtained in clinic to determine whether or not active PDR is present. Standard cameras are not able to image the whole retina. It is possible, however, to obtain several images to capture the appearance of the centre, superior and inferior parts of the retina; the retinal periphery cannot be imaged with standard cameras. In recent years new ultra-wide field imaging has become available, allowing imaging of the entire retina. This technology may be, thus, preferable, and it may be easier for patients and reduce the time required to obtain images.

Given the high number of people with DMO and PDR, the need for patients to be seen at short follow-up intervals, the need for frequent treatments and the requirement for long-term follow-up, there is a very large workload in HES related to DMO/PDR. This is making it difficult for the NHS to cope with the demand, in particular, due to shortage of ophthalmologists. This is only expected to get worse given the increasing prevalence of diabetes. Identifying new ways of increasing the NHS capacity and efficiency without compromising quality of care would greatly benefit the NHS.

The purpose of EMERALD is to determine whether successfully treated patients with DMO/PDR could be followed without a face-to-face examination by an ophthalmologist. EMERALD will evaluate a new care pathway which will include multimodal retinal imaging and separate image assessment by trained ophthalmic graders. This new pathway will be compared to the current standard care pathway which is, for DMO, having an ophthalmologist evaluating patients in clinic by slit-lamp biomicroscopy and with access to SD-OCT images and for PDR evaluating patients in clinic by slit-lamp biomicroscopy. EMERALD will determine how accurate the new pathway is at determining which patients have active or inactive disease when compared with the standard of care and its costs and acceptability by patients and health professionals.

Methods and analysis

1. Study design and setting

Prospective, diagnostic study of patients with DMO, PDR, or both, who had been previously successfully treated and who, at the time of enrolment in the study, may have active or inactive disease (both are required to evaluate the diagnostic performance of the new pathway).

EMERALD has a case-referent cross-sectional diagnostic study design with both sampling (selection) of patients and data collection carried out prospectively (18). This approach provides both a cost-efficient study design and low risk of bias in terms of diagnostic accuracy (19).

EMERALD is set within specialised HES in the UK. All participating centres have extensive experience with the management of DMO and PDR.

For peer review only - http://bmjopen.bmj.com/site/about/guidelines.xhtml

1.1. Patient and Public Involvement

A Patient and Public Involvement (PPI) group was established early on in the study, at the stage of trial conception. The PPI group confirmed the research question was important to patients, the tests to be undertaken for the purpose of the study were adequate and feasible to be performed in the clinical setting, and the outcomes measured relevant to them. The PPI group provided essential input to the patient related materials produced for the study, including the patient information sheet and consent form. Furthermore, patient views on the pathways of care investigated will be evaluated specifically in the study (see Focus Group Discussions section, below). The PPI group will be actively involved in the dissemination of the results. Patients were not involved in the recruitment of participants in the study.

2. Participants: Eligibility criteria

Inclusion Criteria:

Adults (18 years of age or older) with type 1 or 2 diabetes with previously successfully treated DMO and/or PDR in one or both eyes and in whom, at the time of enrolment in the study, DMO and/or PDR may be active or inactive. Patients can be recruited only once.

Active DMO is defined as CRT of > 300 microns and/or presence of intraretinal/subretinal fluid on SD-OCT due to DMO.

Inactive DMO is defined as no intraretinal/subretinal fluid.

Active PDR is defined by the presence of sub-hyaloid/vitreous haemorrhage and/or active new vessels (new vessels with lack of fibrosis on them)

Inactive PDR is defined by the lack of preretinal/vitreous haemorrhage and lack of active new vessels.

Exclusion Criteria:

1) Unable to provide informed consent
2) Patients who do not read, speak or understand English.

3. Outcome measures

Primary outcome:

• Sensitivity of the new pathway (ophthalmic grader pathway) in detecting active DMO/PDR, using the standard care pathway as the reference standard.

Secondary Outcomes:

For peer review only - http://bmjopen.bmj.com/site/about/guidelines.xhtml

• Specificity, concordance (agreement) between new pathway (ophthalmic grader pathway) and the standard care pathway, positive and negative likelihood ratios
• Cost-effectiveness
• Acceptability
• Proportion of patients requiring subsequent full clinical assessment
• Proportions of patients unable to undergo imaging, with inadequate quality images or indeterminate findings.

4. Study Procedures and Schedule of Assessments

Ophthalmic Grader Pathway and Standard Care Pathway

In EMERALD a new clinical care pathway (Figure 1), the ophthalmic grader pathway, will be evaluated and compared with the current standard of care.

If this new pathway were to be used in clinical practice, patients would undergo multimodal retinal imaging with subsequent review by trained ophthalmic graders. If active disease were to be detected or if the ophthalmic grader were to be uncertain as to whether or not there was active DMO/PDR present, the patient would be referred to an ophthalmologist for a full assessment. If the patient were to be stable (i.e. inactive DMO/PDR) the patient would remain under this model of surveillance with a pre-determined interval.

For the purpose of EMERALD, all patients will go through the standard care pathway which is: 1) for DMO: ophthalmologist evaluating patients in clinic by slit-lamp biomicroscopy and with access to SD-OCT images, which are routinely obtained for the evaluation of DMO in clinic; 2) for PDR: ophthalmologists evaluating patients in clinic by slit-lamp biomicroscopy.

Selection of Ophthalmic Graders and Training

Currently, ophthalmic photographers/imaging technicians obtain images and interpret them routinely, but make no decisions on the care of patients. In ophthalmic services, there are also ophthalmic graders that have been trained to interpret findings on fundus images for the purpose of undertaking DR screening.

For EMERALD the ophthalmic graders at each participating site will be selected as follows. First, local principal investigators (PIs) would provide names of individuals they believe have experience obtaining and/or grading images of patients with DMO and PDR; these individuals would be also confirming their interest and willingness to participate in EMERALD. It is possible that some ophthalmic graders selected for EMERALD will be already involved in the grading of images for DR screening.

Graders identified by the PIs will be asked to fill out questionnaires detailing their experience imaging/grading DMO/PDR, recognising features of DMO/PDR, and whether they feel confident identifying DMO on SD-OCT and new vessels on fundus images. Graders stating they do not have experience imaging/grading DMO/PDR and/or those stating they could not recognise features of DMO/PDR will not be invited to take part in EMERALD as graders.

For peer review only - http://bmjopen.bmj.com/site/about/guidelines.xhtml

Formal training, during a training meeting(s) in which features of active/inactive DMO/PDR will be reviewed and discussed and where extensive clinical examples will be presented, will be provided to all EMERALD ophthalmic graders prior to the initiation of the study, as described in the EMERALD Study Manual.

To ensure graders selected will be in a position to undertake the task of grading images, all potential graders will be required to take a test involving the reading of OCTs and fundus images. Only those graders who reach a minimum of 80% of correct answers to detect presence of DMO or active PDR, when present, will be invited to act as graders. Graders will be allowed to undergo further training and take the test a second time but if the minimum of correct answers is not reached they will not be able to act as graders.

A web-based teaching module with examples of DMO/PDR will be prepared so that graders can access it to consolidate their knowledge. Clear guidelines on when patients would need to be referred for an assessment by an ophthalmologist will be also given.

Schedule of Assessments for EMERALD

Patient flow and procedures undertaken in EMERALD have been summarised in Figure 2.

Written informed consent will be obtained by ophthalmologists/deputes from all participants prior to any study procedures being undertaken.

The following information obtained during the standard care pathway will be recorded in the Case Report Forms (CRFs):

- Medical and ophthalmic history.
- Best-corrected visual acuity.
- Whether there was active or inactive DMO / PDR and whether or not there were features that could make imaging difficult (e.g. small pupil).
- Details on presence/absence and location of active/inactive new vessels in the disc and elsewhere in the retina and/or pre-retinal haemorrhage/vitreous haemorrhage
- Presence of any other co-existent eye disease noted.
- Information on the proposed plan for the patient.

Patients will undergo ETDRS 7 field and ultra-wide field imaging, obtained following the guidelines set in the EMERALD Study Manual and will complete EQ-5D 5L, NEI VFQ-25 and VisQoL questionnaires.

Patients willing to participate in the Focus Group Discussions will be consented; a group of them will be contacted at a later date for this purpose (see below).

Fundus photographs and SD-OCTs will be anonymised and transferred to Queens' University Belfast (QUB) reading centre where they will be uploaded in an electronic website developed for the study; these anonymised images will be then made

For peer review only - http://bmjopen.bmj.com/site/about/guidelines.xhtml

accessible to the ophthalmic graders (and ophthalmologists determining the "enhanced reference standard" for PDR, see below) for the purpose of grading.

Ophthalmic graders will determine (and record in the appropriate CRF):
- Whether there is active/inactive DMO/PDR (or if unsure)
- Location of new vessels
- Whether patient could continue review in the ophthalmic grader pathway or requires assessment by an ophthalmologist and the reasons why.

Enhanced Reference Standard

The reference standard for PDR (ophthalmologist evaluating patients in clinic by slit-lamp biomicroscopy) will be used for the primary analysis. The reference standard for PDR, however, could potentially be improved. There is a possibility that new vessels (indicative of PDR) may not be seen by the ophthalmologist on slit-lamp biomicroscopy but could be detected in a fundus photograph. In order to determine the impact of this potential event EMERALD will also evaluate an alternative "enhanced" reference standard which will consist of the ophthalmologist assessment supplemented by the evaluation of the fundus images (7 field ETDRS and ultra-wide field fundus images) done by an ophthalmologist. This reading by the ophthalmologist of the fundus images will be done only after the reference standard has been set, to ensure it will not affect or influence the reference standard. If either, slit-lamp biomicroscopy, 7 field ETDRS or ultra-wide angle fundus images detect active PDR, the patient will be considered to have "active" PDR under this "enhanced" reference standard. This information will be recorded in the appropriate CRF. The PDR status based upon the enhanced reference standard will be used in a sensitivity analysis of the new pathway's diagnostic accuracy.

Masking

Ophthalmic graders will be masked to the reference standard; to ensure this, they will not interpret images from patients recruited at their own centre and will not have access to results of the reference standard. They will not read 7 field ETDRS and wide-angle fundus images or SD-OCTs of the same eye either, to ensure their reading of one imaging technology will not influence their reading of the other.

Ophthalmologists doing the standard of care evaluation will also be masked to the findings/decisions made by the ophthalmic graders (who will be reviewing the images at a later date).

Ophthalmologists determining the "enhanced" reference standard will be masked in the same manner as the ophthalmic graders.

Patients will also be masked to findings/decisions made by the ophthalmic graders (these will not be available at the time of the study's clinical visit).

5. Data collection and quality checks

For peer review only - http://bmjopen.bmj.com/site/about/guidelines.xhtml

CRFs will be used to collect data. Monitoring during the study will check the accuracy of entries on CRF's against source documents, adherence to protocol, procedures and to the International Conference of Harmonisation Good Clinical Practice (ICH-GCP) guidelines and regulatory requirements. Monitoring visits will be undertaken by a monitor from the Northern Ireland Clinical Trials Unit (NICTU). To ensure accurate, complete and reliable data are collected, the Chief Investigator and the NICTU will provide training to site staff.

Study data will be entered onto a web-based Clinical Trial Database by NICTU personnel and processed electronically. Data quality control checks will be carried out by a data manager to ensure accuracy; data errors will be documented in Quality Control Reports with corrective actions implemented. Data validation will be implemented and discrepancy reports will be generated following data entry to identify discrepancies such as out of range, inconsistencies or protocol deviations based on data validation checks programmed in the clinical trial database.

6. Sample size

The sample size was determined upon the basis of setting a target of the number of reactivated (active) DMO and PDR patients which would enable sensitivity to be tested against a pre-specified target level of 80% (20). This level was considered the minimum acceptable level for the new pathway (ophthalmic grader pathway) to be clinically viable. A lower specificity is considered acceptable and a target of 65% for specificity was used to confirm sufficiency of the sample size for assessing specificity. To detect the sensitivity of the new pathway (photographer/imaging technician pathway) with 80% and 90% power (10% and 12% higher than the 80% minimal target set) would require 89 participants with each DMO/ PDR who have reactivated (active DMO/PDR), with 2-sided 5% significance level (21). Ninety-three participants who have not reactivated would enable a specificity 15% (10%) higher than the 65% target to be detected with 90% power. A 95% confidence interval for photographer sensitivity and specificity would have a confidence interval (Wilson method) with a width of 10-20% depending on the observed level (22). Allowing for 10% missing/indeterminate results, 104 individuals who have re-activated and 104 who have not, are required (208 for each, DMO and PDR) which leads to a need for a maximum of 416 participants in the study overall; some participants may have both DMO and PDR contributing to both DMO and PDR targets.

7. Data Analysis Plan

Outcomes for DMO and PDR will be assessed in two separate analyses. Participants will be categorised as having active, inactive, or no DMO/PDR according to the diagnosis established at the standard care pathway at the person level (i.e. using data from both eyes where appropriate). Active and inactive DMO/PDR will be subcategorised as previously successfully treated or not. Those which had previously successfully treated DMO/PDR constitute "eligible" patients for the new pathway. This person based assessment reflects the consequences of the clinical decision. The diagnostic performance of the new pathway will be quantified by assessing against the standard care pathway. Reflecting how the new pathway

For peer review only - http://bmjopen.bmj.com/site/about/guidelines.xhtml

would function in practice, an “unsure” classification or an “active” classification will both require an examination by an ophthalmologist under the main analyses.

A number of sensitivity analyses will be carried out. These will include 1) an assessment of the impact of the “unsure” ophthalmic classification upon the diagnostic performance; 2) of using the ophthalmologist’s decision to do further treatment rather than presence of active disease, given than in some patients with active disease the ophthalmologist may still consider observation if only very mild disease is present, 3) detection of disease in more severe cases (e.g. high risk PDR), and 4) of the diagnostic performance within routine NHS clinics versus those set-up specifically for this research study. In addition, for PDR, a sensitivity analysis will assess the diagnostic performance of the ophthalmic grader against the “enhanced” reference standard

The impact of using ultra-wide field or 7 field ETDRS images on the diagnostic performance of the new pathway will be assessed also under the principal analyses for PDR using both the standard and the enhanced reference standard.

Furthermore, secondary analyses will include 1) an evaluation based on eye level data, 2) and analysis that will include all patients (i.e. including those with no disease), 3) an assessment of using tests of both DMO and PDR with regards to an overall referral status, and 4) the additional use of visual acuity as a proxy to detect active disease. We will quantify the proportion of patients identified by an ophthalmologist as having other eye comorbidities (e.g. epiretinal membrane, glaucoma, others) and will explore in relevant analyses their influence on the findings.

Sensitivity, specificity, positive and negative likelihood ratios will be calculated [with appropriate 95% confidence intervals (CIs)] for the alternative strategy using the current standard of care pathway findings as the reference standard. Agreement (concordance) between the new and current standard of care pathways will also be calculated (with 95% Wilson CI) (22). The difference in sensitivity and specificity between ultra-wide field and 7 field ETDRS images assessed by the ophthalmic graders will be compared with corresponding 95% CIs produced using Newcombe’s method for paired data (23).

The proportion of patients requiring subsequent full clinical assessment or unable to undergo assessments, with inadequate quality images or indeterminate findings will be calculated for the alternative pathway with corresponding CIs. All analyses will be carried out using STATA 15 (24).

A detailed Statistical Analysis Plan (SAP) will be written by the trial statistician prior to the final analysis.

8. Health Economic Analysis

This analysis will take into account: 1) sensitivity and specificity of the ophthalmic grader pathway for both DMO and PDR; 2) whether this new pathway detects any PDR missed by the current standard of care; and 3) relative costs.

In the ophthalmic grader pathway, patients will be in one of four groups depending on the decisions made by these staff after reading the images: 1) true negative – no treatment required and patient will return for follow-up at the usual interval; 2) true positive – referred for ophthalmic assessment and treatment as required; 3) false negative – patient who may come to harm by visual loss; 4) false

For peer review only - http://bmjopen.bmj.com/site/about/guidelines.xhtml

positive – patient will be referred to the ophthalmologist but will not require treatment; these patients will not come to harm apart from possible anxiety and inconvenience, but will consume ophthalmologist time.

Scores obtained in the health-related quality of life questionnaire (EQ-5D-5L) and visual function questionnaires (NEI VFQ-25 and Vis-QoL) will provide utility data for different health states. Resource use data will be collected to explore the costs of delivering the standard care pathway and the ophthalmic grader pathway and to find the key cost drivers. This will mainly consist of staff costs. Costs of 7-field ETDRS images will be compared with those of ultra-wide angle imaging.

If the sensitivity and specificity of the new and standard pathways were exactly the same there would be no quality-adjusted life year (QALY) differences, though there might be some disutility from process changes (i.e. if one pathway caused more anxiety than the other). The key gain would be ophthalmologist time freed for other activities. The real benefits might be reduction in waiting times and earlier treatment of other patients leading to QALY gains for them. Such benefits would be difficult to estimate and the simplest measure would be ophthalmologist sessions/days released for other activities. However, we will identify ways in which time released would be used. An underlying assumption to be checked is that the cost of assessment by the ophthalmic graders is less than that of the ophthalmologists. Both pathways would require nurse or optometrist time for checking visual acuity, as done in routine clinical practice. The costs of the image-based pathways will include both time for taking and reading images, using both conventional and ultra-wide field cameras. If there was marginal loss in sensitivity from the new pathway the consequences could be visual loss before next visit was due, or detection at next visit (with or without visual loss occurring) followed by possibly later than optimal treatment. Both could have disutilities.

It should be noted that assessment in clinical practice is repeated over time so active disease missed at one assessment might be picked up at the next. Given the cross-sectional design of EMERALD and the fact that all patients will undergo the standard care pathway, we will not be able to assess the disutility of any visual changes in patients recruited.

We will develop a Markov model using data from both this study (the EQ-5D 5L data for different states) and published studies on progression, so analysis of the effect of a reduced sensitivity will include, for DMO and PDR separately: 1) probability of progression before next visit, 2) probability that this would lead to irreversible visual loss and if so, how much; 3) if there was irreversible visual loss, the resulting disutility and QALY loss.

Specificity would be the determining factor in savings in ophthalmologist time: the poorer the specificity the lower the savings. In the PDR group, ultra-wide field imaging will be compared with both, standard care (ophthalmologist evaluating patients by slit-lamp biomicroscopy) and 7-field ETDRS to assess the cost-effectiveness of this more recently introduced imaging modality.

Any future costs and benefits will be discounted at 3.5%. NHS and personal social services perspective will be adopted. The model will be populated by cost, sensitivity and specificity data from the study and by estimates of progression, effectiveness of treatment (prompt and delayed), quality of life and future costs from published literature and expert opinion. Results will be expressed as cost per QALY gained. Appropriate sensitivity analyses will be conducted to assess robustness of results. Probabilistic sensitivity analyses will be undertaken to explore uncertainty in

For peer review only - http://bmjopen.bmj.com/site/about/guidelines.xhtml

model parameters and to allow presentation of cost-effectiveness acceptability curves.

9. Focus Groups Discussions: Assessment of Acceptability of the New Care Pathway

The acceptability of the ophthalmic graders pathway will be evaluated through focus group discussions. Patients' views on the acceptability of the proposed new pathway are essential if this were to be incorporated into clinical practice. Focus group discussions are particularly useful to help identify issues that resonate with lay people in matters of health care and have been widely used in health services research; focus group discussions reach the parts that other methods cannot (25). Their use in this study will enable us to acquire data on a full range of issues – some of which are unlikely to have been anticipated by professionals. The sample frame is designed to include consenting participants drawn from different UK areas and different age groups. Group discussions will be facilitated by a trained researcher. Discussions will be audio-recorded and later transcribed for analysis.

Informed consent will be obtained from participants prior to the focus group discussions. Participants consenting to take part will be approached at a later date via letter/phone call to inform them about the date/location/time of the meeting.

EMERALD will also examine the acceptability of the new pathway to health professionals. For this purpose, a small number of focus groups will be conducted involving photographer/imaging technicians/graders and ophthalmologists, all recruited from staff at participating sites.

The data from the focus groups will be analysed by the use of simple content analysis strategies. The focus of the analysis will be on 'acceptability' of the new alternative pathway and factors that might facilitate or impede such acceptability (26).

Ethics and Dissemination

Ethics approval was obtained from the Office for Research Ethics Committees Northern Ireland (ORECNI - 17/NI/0124).

The study is conducted in compliance with the protocol approved by the Research Ethics Committee (REC). Changes to the protocol may require REC approval prior to implementation. The NICTU in collaboration with the sponsor will submit all protocol modifications to the REC for review in accordance with the governing regulations.

Serious Adverse Events (SAE) will be recorded and reported to ORECNI.

In order to maintain participant's confidentiality, a unique study identification number will be used for each participant. Study reports and communication regarding the study will identify the patients by their assigned unique trial identifier. Computers where information will be stored will be password protected.

A report containing the methodology and results of EMERALD will be published as a Health Technology Assessment monograph, freely accessible via the NIHR HTA web site. The Royal College of Ophthalmologist will be contacted when the study is completed to allow the study findings to be incorporated in future Diabetic

For peer review only - http://bmjopen.bmj.com/site/about/guidelines.xhtml

Retinopathy guidelines. Findings will be published in national/international peer reviewed journals and presented at national/international meetings and patient groups.

References

[1] Liew G, Michaelides M, Bunce C. A comparison of the causes of blindness certifications in England and Wales in working age adults (16-64 years), 1999-2000 with 2009-2010. *BMJ Open* 2014, 4:e004015.

[2] Zheng Y, He M, Congdon N. The worldwide epidemic of diabetic retinopathy. *Indian J Ophthalmol* , 2012;60:428-31.

[3] Minassian DC, Owens DR, Reidy A. Prevalence of diabetic macular oedema and related health and social care resource use in England. *Br J Ophthalmol*. 2012;96:345-9.

[4] Yau JW, Rogers SL, Kawasaki R, et al: Meta-Analysis for Eye Disease (META-EYE) Study Group. Global prevalence and major risk factors of diabetic retinopathy. *Diabetes Care*, 2012;35:556-64.

[5] https://www.diabetes.org.uk.

[6] www.nice.org.uk/guidance/ta274.

[7] www.nice.org.uk/guidance/ta346.

[8] https://www.nice.org.uk/guidance/ta301.

[9] https://www.rcophth.ac.uk/wp-content/uploads/2014/12/2013-SCI-301-FINAL-DR-GUIDELINES-DEC-2012-updated-July-2013.pdf.

[10] Keenan TD, Johnston RL, Donachie PH et al. United Kingdom National Ophthalmology Database Study: Diabetic Retinopathy; Report 1: prevalence of centre-involving diabetic macular oedema and other grades of maculopathy and retinopathy in hospital eye services. *Eye* , 2013;27:1397-404.

[11] Diabetic Retinopathy Clinical Research Network, Elman MJ, Aiello LP, Beck RW, et al. Randomized trial evaluating ranibizumab plus prompt or deferred laser or triamcinolone plus prompt laser for diabetic macular edema. *Ophthalmology* 2010;117:1064- 1077.

[12] Mitchell P, Bandello F, Schmidt-Erfurth U,et al. The RESTORE study: ranibizumab monotherapy or combined with laser versus laser monotherapy for diabetic macular edema. *Ophthalmology* 2011;118:615-25.

[13] Nguyen QD, Brown DM, Marcus DM et al. Ranibizumab for diabetic macular edema: results from 2 phase III randomized trials: RISE and RIDE. *Ophthalmology* 2012;119:789-801.

For peer review only - http://bmjopen.bmj.com/site/about/guidelines.xhtml

For peer review only

[14] Korobelnik JF, Do DV, Schmidt-Erfurth U, et al. Intravitreal aflibercept for diabetic macular edema. *Ophthalmology* 2014;121:2247-2254.

[15] Diabetic Retinopathy Clinical Research Network, Wells JA, Glassman AR, Ayala AR, et al. Aflibercept, bevacizumab, or ranibizumab for diabetic macular edema. *New Engl J Med* 2015;372:1193-203.

[16] Virgili G, Menchini F, Casazza G, et al. Optical coherence tomography (OCT) for detection of macular oedema in patients with diabetic retinopathy. *Cochrane Database Syst Rev.* 2015;1:CD008081.

[17] Olson J, Sharp P, Goatman K, et al. Improving the economic value of photographic screening for optical coherence tomography-detectable macular oedema: a prospective, multicentre, UK study. *Health Technol Assess*. 2013;17:1-142.

[18] Knottnerus JA, Muris JW. Assessment of the accuracy of diagnostic tests: the cross-sectional study. *Journal of Clinical Epidemiology* 2003;56:1118–1128.

[19] Whiting PF, Rutjes AWS, Westwood ME, et al. QUADAS-2: A Revised Tool for the Quality Assessment of Diagnostic Accuracy Studies. *Ann Intern Med* 2011;155:529-536.

[20] Obuchowski NA. Sample size calculations in studies of test accuracy. *Statistical Methods in Medical Research* 1998;7:371-392.

[21] Silva PS, Cavallerano JD, Tolson AM, et al. Real-Time Ultrawide Field Image Evaluation of Retinopathy in a Diabetes Telemedicine Program. *Diabetes Care*, 2015;38:1643-9.

[22] Piegorsch WW. Sample sizes for improved binomial confidence intervals. *Computational Statistics & Data Analysis* 2004;46:309 – 316.

[23] Newcombe RG. Improved confidence intervals for the difference between binomial proportions based on paired data. *Stats in Medicine* 1998;17:2635-2650.

[24] StataCorp. 2017. Stata Statistical Software: Release 14. College Station, TX: StataCorp LP.

[25] Kitzinger, J. (1995). Qualitative Research: Introducing focus groups, *BMJ*;311:299-302.

[26] Prior, L. Content Analysis. In, P. Leavy. Ed. *The Oxford Handbook of Qualitative Research Methods*. 2014; Oxford Library.

For peer review only - http://bmjopen.bmj.com/site/about/guidelines.xhtml

Legends for Figures

Figure 1: Ophthalmic Grader's Pathway, Summary.

Figure 2: Study Flowchart.

For peer review only

Authorship policy: An author is considered someone who has made a substantive intellectual contribution to the study and the relevant report.

EMERALD STUDY GROUP: Ahmed Saad, James Cook University Hospital, South Tees Hospitals NHS Foundation Trust; Augusto Azuara-Blanco, Queen's University and Royal Victoria Hospital, Belfast H&SC Trust; Caroline Styles, Queen's Margaret Hospital, Fife; Christine McNally, Andrew Jackson and Rachael Rice, Northern Ireland Clinical Trials Unit; Clare Bailey, Bristol Eye Hospital, University Hospitals Bristol NHS Foundation Trust; Danny McAuley, Queen's University and Royal Victoria Hospital, Belfast H&SC Trust; David H Steel, Sunderland Eye Infirmary, City Hospitals Sunderland NHS Foundation Trust; Faruque D Ghanchi, Bradford Teaching Hospitals NHS Trust; Geeta Menon, Frimley Park Hospital NHS Foundation Trust; Haralabos Eleftheriadis, Kings College Hospital NHS Foundation Trust; Hema Mistry, Warwick University; Jonathan Cook and William Sones, Centre for Statistics in Medicine, University of Oxford; Lindsay Prior, Centre for Public Health, Queens University, Belfast; Nachiketa Acharya, Sheffield Teaching Hospitals NHS Foundation Trust; Noemi Lois, Queen's University and Royal Victoria Hospital Belfast H&SC Trust; Norman Waugh, Warwick University; Rachael Rice, Northern Ireland Clinical Trials Unit; Samia Fatum, John Radcliffe Hospital, Oxford University Hospitals NHS Foundation Trust; Sobha Sivaprasad, Moorfields Eye Hospital NHS Foundation Trust; Stephen Aldington and Peter H Scanlon, Gloucestershire Hospitals NHS Foundation Trust; Tariq M Aslam, Manchester Royal Eye Hospital, Central Manchester University Hospitals NHS Foundation Trust; Victor Chong, Royal Free Hospital NHS Foundation Trust, London.

Authors' Contributions: NL conceived the study and drafted the protocol which was refined with input from JAC, SA, NW, HM, WS, DM, TMA, CB, VC, FDG, PHS, SS, DHS, CS, LP and AAB; JAC and NL determined the sample size for the study and JAC, WS and NL determined the statistical analysis plan. NW and HM planned the cost-effectiveness evaluation. LP designed and planned the focus group discussions and will carry them out. CM, RR and AJ provided management coordination and data management to the study. NL, AS, CB, DHS, FDG, GM, HE, NA, SF, SS, SA, PHS, TMA will recruit and evaluate patients for the study. All authors reviewed the manuscript, provided input to it and approved the final submitted version.

Clinical sites participating in recruitment: Bradford Royal Infirmary, Bradford Teaching Hospitals NHS Foundation Trust; Bristol Eye Hospital, University Hospitals Bristol NHS Foundation Trust; Frimley Park Hospital NHS Foundation Trust; Gloucestershire Royal Hospital, Gloucestershire Hospitals NHSF Trust; James Cook University Hospital, South Tees Hospitals NHS Foundation Trust;Kings College Hospital NHS Foundation Trust; Manchester Royal Eye Hospital, Central Manchester University Hospitals NHS Foundation Trust; Moorfields Eye Hospital NHS Foundation Trust; John Radcliffe Hospital, Oxford University Hospitals NHS Foundation Trust; Queen Margaret Hospital, Fife; Royal Victoria Hospital, Belfast H&SC Trust; Sheffield Teaching Hospitals NHS Foundation Trust; Sunderland Eye Infirmary, City Hospitals Sunderland NHS Foundation Trust.

EMERALD is currently recruiting. The first trial participant was recruited in October 2017.

For peer review only - http://bmjopen.bmj.com/site/about/guidelines.xhtml

Acknowledgement: To optometrists, research coordinators, research nurses, ophthalmic technicians and ophthalmic photographers contributing to EMERALD at each of the participating sites. To all patients participating in EMERALD, to those who were part of the PPI group and to Diabetes UK, Northern Ireland. To Janice Bailie, Alison Murphy and Lynn Murphy for their support, help and assistance with this diagnostic study and the UK Ophthalmology Clinical Research Network, Maurice O'Kane, Julie Silvestri, Jonathan Jackson, Paul Biagioni and the Northern Ireland Ophthalmology Clinical Research Network for their support to EMERALD.

Trial Management Group: Prof Noemi Lois (Chief Investigator), Prof. Augusto Azuara-Blanco, Mr Steve Aldington, Dr Danny McAuley, Mr Peter Scanlon, Mr Lindsay Prior, Mrs Clare Newall, Mrs Michelle McGaughey, Mrs Christine McNally, Miss Rachael Rice, Mr Andrew Jackson, Mr Jonathan Cook, Mr William Sones, Prof Norman Waugh, Dr Hema Mistry, Mr Mark Wilson, Mrs Nuala Hannaway, Mrs Catherine Campbell.

Trial Steering Committee: Mr John Norrie (Chair); Mr David Owens; Mrs Florence Findlay-White; Mr Jonathan Cook; Mr William Sones; Mr Winfried Amoaku; Miss Yemisi Takwoingi.

The roles and responsibilities of the TSC can be found in https://www.nihr.ac.uk/funding-and-support/documents/funding-for-research-studies/how-to-apply/NETSCC_Project_Oversight_Groups_Guidance.pdf

A study Data and Ethics Monitoring Committee was not deemed necessary given the extremely low risk of this diagnostic study.

Trial Sponsor: The Belfast Health and Social Care Trust. Belfast, NI. Contact person: Ms Alison Murphy; alison.murphy@belfasttrust.hscni.net.

Contact for public queries: EMERALD@nictu.hscni.net

Funding Statement

This project was funded by the NIHR [Health Technology Assessment programme] (project number 13/142/04).

The views expressed are those of the authors and not necessarily those of the NHS, the NIHR or the Department of Health and Social Care.

Neither the Sponsor nor the Funder had any role on the study design; collection, management, analysis, and interpretation of data; writing of this manuscript or in the decision to submit this manuscript for publication.

Competing Interests Statement

For peer review only

Noemi Lois: None; Norman Waugh: None; Augusto Azuara-Blanco: None; Hema Mistry: None; Danny McAuley: None; Tariq Aslam: None; Clare Bailey: has been ad-hoc advisor for Alcon, Bayer, Novartis, Alimera sciences and Allergan; Victor Chong is a part time employee of Boehringer Ingelheim International GmBH (BII), Germany; this study, however, is not being undertaken as part of the employment with BII and, thus, the content of this manuscript is not endorsed by BII. Victor Chong has received also speaker fees from Quantel Medical, France; Sobha Sivaprasad has received research grants, travel feeds and attended advisory board meetings of Novartis, Bayer, Roche, Allergan, Heidelberg Engineering, Optos Plc an Boehringer Ingleheim; David Steel; David Steel acted as Consultant to Alcon, attended advisory boards for Novartis and Bayer and received research funding from Bayer and Alcon. Peter Scanlon has attended Advisory Boards for Allergan, Roche, Boehringer and Bayer; his department has received Educational, Research or Audit Grants from Allergan, Novartis and Bayer.

Data Access

The Chief investigator has access to the trial data and will act as a data guardian.

Word Count: 4701, excluding abstract & references.

For peer review only

Ophthalmic Grader Pathway

- Images will be obtained from both eyes by an ophthalmic photographer/imaging technician, including:
    - Spectral Domain Optical Coherence Tomography (SD-OCT)
    - Early Treatment Diabetic Retinopathy Study (ETDRS) 7 field non-stereoscopic fundus photographs
    - Ultra-wide angle fundus images
- Images will be read by trained ophthalmic graders
    - Presence/absence of active DMO/PDR determined

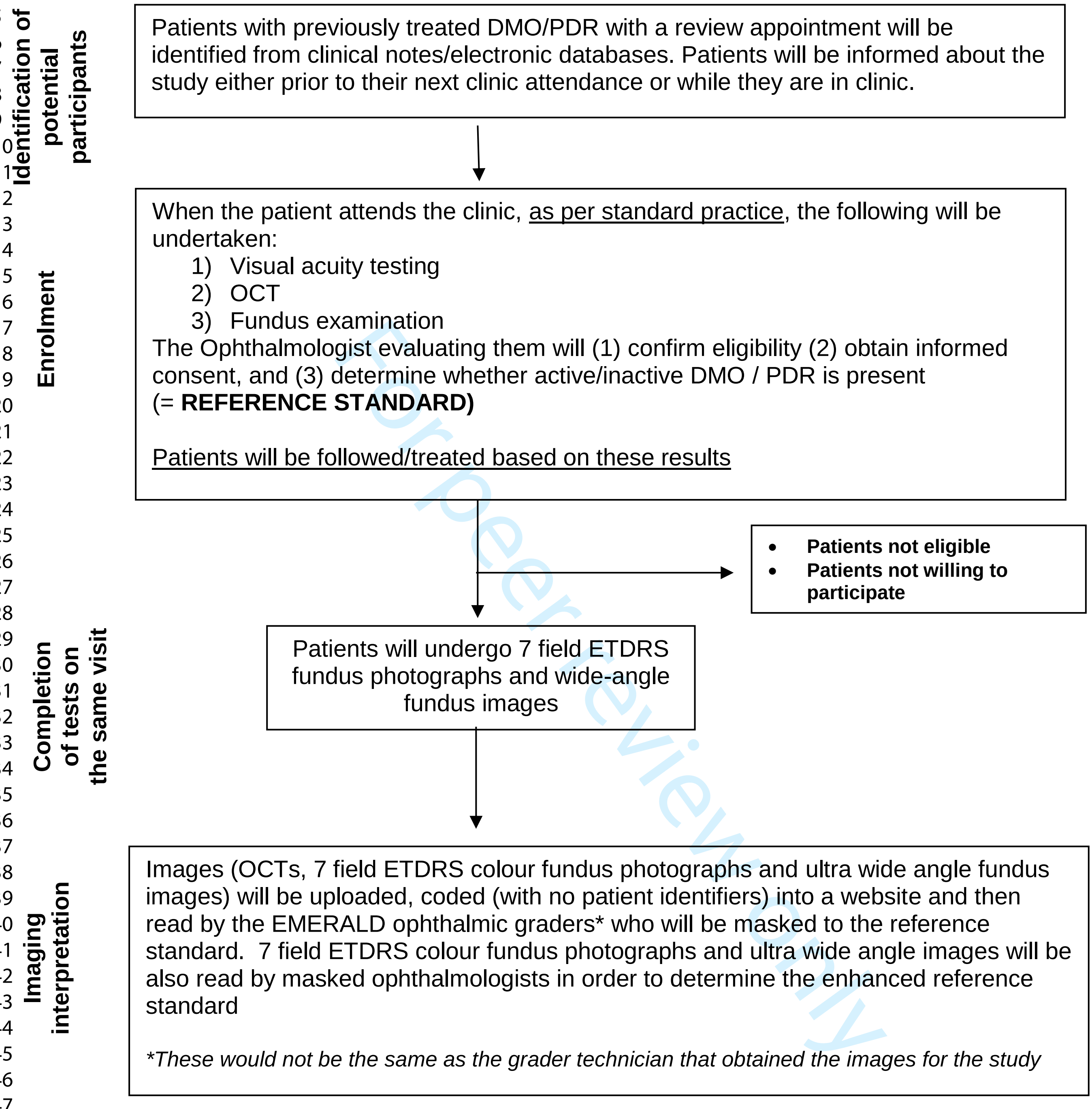

DMO = diabetic macular oedema; PDR = proliferative diabetic retinopathy; OCT = optical coherence tomography; ETDRS = early treatment diabetic retinopathy study

For peer review only

Reporting checklist for protocol of a clinical trial.

Based on the SPIRIT guidelines.

Instructions to authors

Complete this checklist by entering the page numbers from your manuscript where readers will find each of the items listed below.

Your article may not currently address all the items on the checklist. Please modify your text to include the missing information. If you are certain that an item does not apply, please write "n/a" and provide a short explanation.

Upload your completed checklist as an extra file when you submit to a journal.

In your methods section, say that you used the SPIRIT reporting guidelines, and cite them as:

Chan A-W, Tetzlaff JM, Altman DG, Laupacis A, Gøtzsche PC, Krleža-Jerić K, Hróbjartsson A, Mann H, Dickersin K, Berlin J, Doré C, Parulekar W, Summerskill W, Groves T, Schulz K, Sox H, Rockhold FW, Rennie D, Moher D. SPIRIT 2013 Statement: Defining standard protocol items for clinical trials. Ann Intern Med. 2013;158(3):200-207

For peer review only

		Reporting Item	Page Number
Title	#1	Descriptive title identifying the study design, population, interventions, and, if applicable, trial acronym	1
Trial registration	#2a	Trial identifier and registry name. If not yet registered, name of intended registry	1

Trial registration: data set	#2b	All items from the World Health Organization Trial Registration Data Set	1
Protocol version	#3	Date and version identifier	1
Funding	#4	Sources and types of financial, material, and other support	17
Roles and responsibilities: contributorship	#5a	Names, affiliations, and roles of protocol contributors	1, 16
Roles and responsibilities: sponsor contact information	#5b	Name and contact information for the trial sponsor	17
Roles and responsibilities: sponsor and funder	#5c	Role of study sponsor and funders, if any, in study design; collection, management, analysis, and interpretation of data; writing of the report; and the decision to submit the report for publication, including whether they will have ultimate authority over any of these activities	17
Roles and responsibilities: committees	#5d	Composition, roles, and responsibilities of the coordinating centre, steering committee, endpoint adjudication committee, data management team, and other individuals or groups overseeing the trial, if applicable (see Item 21a for data monitoring committee)	17

For peer review only

Background and rationale	#6a	Description of research question and justification for undertaking the trial, including summary of relevant studies (published and unpublished) examining benefits and harms for each intervention	3,4
Background and rationale: choice of comparators	#6b	Explanation for choice of comparators	3,4
Objectives	#7	Specific objectives or hypotheses	3,4
Trial design	#8	Description of trial design including type of trial (eg, parallel group, crossover, factorial, single group), allocation ratio, and framework (eg, superiority, equivalence, non-inferiority, exploratory)	4,5
Study setting	#9	Description of study settings (eg, community clinic, academic hospital) and list of countries where data will be collected. Reference to where list of study sites can be obtained	5
Eligibility criteria	#10	Inclusion and exclusion criteria for participants. If applicable, eligibility criteria for study centres and individuals who will perform the interventions (eg, surgeons, psychotherapists)	5
Interventions: description	#11a	Interventions for each group with sufficient detail to allow replication, including how and when they will be administered	6,7,8

For peer review only

Interventions: modifications	#11b	Criteria for discontinuing or modifying allocated interventions for a given trial participant (eg, drug dose change in response to harms, participant request, or improving / worsening disease)	N/A
Interventions: adherance	#11c	Strategies to improve adherence to intervention protocols, and any procedures for monitoring adherence (eg, drug tablet return; laboratory tests)	N/A
Interventions: concomitant care	#11d	Relevant concomitant care and interventions that are permitted or prohibited during the trial	N/A
Outcomes	#12	Primary, secondary, and other outcomes, including the specific measurement variable (eg, systolic blood pressure), analysis metric (eg, change from baseline, final value, time to event), method of aggregation (eg, median, proportion), and time point for each outcome. Explanation of the clinical relevance of chosen efficacy and harm outcomes is strongly recommended	5,6
Participant timeline	#13	Time schedule of enrolment, interventions (including any run-ins and washouts), assessments, and visits for participants. A schematic diagram is highly recommended (see Figure)	Figures 1,2
Sample size	#14	Estimated number of participants needed to achieve study objectives and how it was determined, including clinical and statistical assumptions supporting any sample size calculations	10

For peer review only

Recruitment	#15	Strategies for achieving adequate participant enrolment to reach target sample size	Figure 2
Allocation: sequence generation	#16a	Method of generating the allocation sequence (eg, computer-generated random numbers), and list of any factors for stratification. To reduce predictability of a random sequence, details of any planned restriction (eg, blocking) should be provided in a separate document that is unavailable to those who enrol participants or assign interventions	N/A
Allocation concealment mechanism	#16b	Mechanism of implementing the allocation sequence (eg, central telephone; sequentially numbered, opaque, sealed envelopes), describing any steps to conceal the sequence until interventions are assigned	N/A
Allocation: implementation	#16c	Who will generate the allocation sequence, who will enrol participants, and who will assign participants to interventions	N/A
Blinding (masking)	#17a	Who will be blinded after assignment to interventions (eg, trial participants, care providers, outcome assessors, data analysts), and how	9,10
Blinding (masking): emergency unblinding	#17b	If blinded, circumstances under which unblinding is permissible, and procedure for revealing a participant's allocated intervention during the trial	N/A

For peer review only

Data collection plan	#18a	Plans for assessment and collection of outcome, baseline, and other trial data, including any related processes to promote data quality (eg, duplicate measurements, training of assessors) and a description of study instruments (eg, questionnaires, laboratory tests) along with their reliability and validity, if known. Reference to where data collection forms can be found, if not in the protocol	8,9,10
Data collection plan: retention	#18b	Plans to promote participant retention and complete follow-up, including list of any outcome data to be collected for participants who discontinue or deviate from intervention protocols	N/A
Data management	#19	Plans for data entry, coding, security, and storage, including any related processes to promote data quality (eg, double data entry; range checks for data values). Reference to where details of data management procedures can be found, if not in the protocol	10
Statistics: outcomes	#20a	Statistical methods for analysing primary and secondary outcomes. Reference to where other details of the statistical analysis plan can be found, if not in the protocol	11,12,13
Statistics: additional analyses	#20b	Methods for any additional analyses (eg, subgroup and adjusted analyses)	11,12,13

For peer review only

Statistics: analysis population and missing data	#20c	Definition of analysis population relating to protocol non-adherence (eg, as randomised analysis), and any statistical methods to handle missing data (eg, multiple imputation)	11,12,13
Data monitoring: formal committee	#21a	Composition of data monitoring committee (DMC); summary of its role and reporting structure; statement of whether it is independent from the sponsor and competing interests; and reference to where further details about its charter can be found, if not in the protocol. Alternatively, an explanation of why a DMC is not needed	17
Data monitoring: interim analysis	#21b	Description of any interim analyses and stopping guidelines, including who will have access to these interim results and make the final decision to terminate the trial	N/A
Harms	#22	Plans for collecting, assessing, reporting, and managing solicited and spontaneously reported adverse events and other unintended effects of trial interventions or trial conduct	N/A
Auditing	#23	Frequency and procedures for auditing trial conduct, if any, and whether the process will be independent from investigators and the sponsor	N/A
Research ethics approval	#24	Plans for seeking research ethics committee / institutional review board (REC / IRB) approval	13

For peer review only

Protocol amendments	#25	Plans for communicating important protocol modifications (eg, changes to eligibility criteria, outcomes, analyses) to relevant parties (eg, investigators, REC / IRBs, trial participants, trial registries, journals, regulators)	13
Consent or assent	#26a	Who will obtain informed consent or assent from potential trial participants or authorised surrogates, and how (see Item 32)	8
Consent or assent: ancillary studies	#26b	Additional consent provisions for collection and use of participant data and biological specimens in ancillary studies, if applicable	N/A
Confidentiality	#27	How personal information about potential and enrolled participants will be collected, shared, and maintained in order to protect confidentiality before, during, and after the trial	10,13
Declaration of interests	#28	Financial and other competing interests for principal investigators for the overall trial and each study site	17,18
Data access	#29	Statement of who will have access to the final trial dataset, and disclosure of contractual agreements that limit such access for investigators	18
Ancillary and post trial care	#30	Provisions, if any, for ancillary and post-trial care, and for compensation to those who suffer harm from trial participation	N/A

For peer review only

Dissemination policy: trial results	#31a	Plans for investigators and sponsor to communicate trial results to participants, healthcare professionals, the public, and other relevant groups (eg, via publication, reporting in results databases, or other data sharing arrangements), including any publication restrictions	13
Dissemination policy: authorship	#31b	Authorship eligibility guidelines and any intended use of professional writers	16
Dissemination policy: reproducible research	#31c	Plans, if any, for granting public access to the full protocol, participant-level dataset, and statistical code	N/A
Informed consent materials	#32	Model consent form and other related documentation given to participants and authorised surrogates	1
Biological specimens	#33	Plans for collection, laboratory evaluation, and storage of biological specimens for genetic or molecular analysis in the current trial and for future use in ancillary studies, if applicable	N/A

Author notes

The SPIRIT checklist is distributed under the terms of the Creative Commons Attribution License CC-BY-ND 3.0. This checklist was completed on 05. September 2018 using http://www.goodreports.org/, a tool made by the [EQUATOR Network]() in collaboration with [Penelope.ai]()

For peer review only
